# Developmentally Sensitive Interaction Effects of Genes and the Social Environment on Total and Subcortical Brain Volumes

**DOI:** 10.1371/journal.pone.0155755

**Published:** 2016-05-24

**Authors:** Jennifer S. Richards, Alejandro Arias Vásquez, Barbara Franke, Pieter J. Hoekstra, Dirk J. Heslenfeld, Jaap Oosterlaan, Stephen V. Faraone, Jan K. Buitelaar, Catharina A. Hartman

**Affiliations:** 1 Department of Cognitive Neuroscience, Donders Institute for Brain, Cognition and Behaviour, Radboud university medical center, Nijmegen, The Netherlands; 2 Karakter Child and Adolescent Psychiatry University Centre, Nijmegen, The Netherlands; 3 Department of Human Genetics, Donders Institute for Brain, Cognition and Behaviour, Radboud university medical center, Nijmegen, The Netherlands; 4 Department of Psychiatry, Donders Institute for Brain, Cognition and Behaviour, Radboud university medical center, Nijmegen, The Netherlands; 5 University of Groningen, University Medical Center Groningen, Department of Psychiatry, Groningen, The Netherlands; 6 Department of Clinical Neuropsychology, VU University Amsterdam, Amsterdam, The Netherlands; 7 Departments of Psychiatry and of Neuroscience and Physiology, SUNY Upstate Medical University, Syracuse, NY, United States of America; 8 K.G. Jebsen Centre for Research on Neuropsychiatric Disorders, University of Bergen, Bergen, Norway; King's College London, UNITED KINGDOM

## Abstract

Smaller total brain and subcortical volumes have been linked to psychopathology including attention-deficit/hyperactivity disorder (ADHD). Identifying mechanisms underlying these alterations, therefore, is of great importance. We investigated the role of gene-environment interactions (GxE) in interindividual variability of total gray matter (GM), caudate, and putamen volumes. Brain volumes were derived from structural magnetic resonance imaging scans in participants with (N = 312) and without ADHD (N = 437) from N = 402 families (age M = 17.00, SD = 3.60). GxE effects between *DAT1*, *5-HTT*, and *DRD4* and social environments (maternal expressed warmth and criticism; positive and deviant peer affiliation) as well as the possible moderating effect of age were examined using linear mixed modeling. We also tested whether findings depended on ADHD severity. Deviant peer affiliation was associated with lower caudate volume. Participants with low deviant peer affiliations had larger total GM volumes with increasing age. Likewise, developmentally sensitive GxE effects were found on total GM and putamen volume. For total GM, differential age effects were found for *DAT1* 9-repeat and *HTTLPR* L/L genotypes, depending on the amount of positive peer affiliation. For putamen volume, *DRD4* 7-repeat carriers and *DAT1* 10/10 homozygotes showed opposite age relations depending on positive peer affiliation and maternal criticism, respectively. All results were independent of ADHD severity. The presence of differential age-dependent GxE effects might explain the diverse and sometimes opposing results of environmental and genetic effects on brain volumes observed so far.

## Introduction

Smaller total brain and subcortical volumes have been linked to various forms of psychopathology including attention-deficit/hyperactivity disorder (ADHD) [1, 2]. Identifying mechanisms underlying brain volume alterations therefore is of great importance. Both genetic and environmental factors play a crucial role in determining interindividual variability in brain architecture [3]. Twin studies have revealed moderate to high heritability estimates for several brain structures (40–97%) [4, 5] and population-based and case-control studies show effects of specific genetic variants in brain volume variation, e.g. [6, 7, 8]. A number of studies have focused on associations with dopamine- and serotonin-related genes. For example, the short allele of a functional promoter polymorphism in the *serotonin transporter gene* (*SLC6A4/ 5-HTT*)—*HTTLPR*—has been related to smaller anterior cingulate gyrus and amygdala volumes in healthy adults [9], smaller frontal cortex in adults with obsessive-compulsive disorder and healthy controls [9, 10], and to smaller caudate, and both smaller and larger hippocampal volumes in adults with major depression [11, 12] and healthy controls [13]. The 10-repeat variant of a variable number tandem repeat (VNTR) polymorphism in the 3’untranslated region (UTR) of the *dopamine transporter gene* (*SLC6A3/DAT1*) has been related to smaller caudate volumes in children with ADHD and controls [14, 15]. Furthermore, variants of a VNTR in exon 3 of the *dopamine receptor D4 gene* (*DRD4*) have been associated with thinner frontal and parietal cortex thickness, and less prefrontal gray matter (GM) volume in children with ADHD and healthy controls as well [14, 16].

Besides genetic factors, environmental influences have also been associated with brain volume changes. So far, most studies have focused on severe negative experiences, such as childhood maltreatment and early life stress [17–21]. However, effects of positive influences, such as maternal warmth or support [22, 23] and, in animal studies, enriched environments [24] have also been reported, thereby broadening the view that only severe negative experiences are associated with brain structures. Nonetheless, studies have reported inconsistent results with findings of both smaller and larger volumes for both positive and negative environmental experiences. These inconsistencies could be due to methodological issues, such as differences in sample characteristics. For example, the majority of studies have used relatively small samples, with most including less than 100 participants (see e.g. [21] for a review). Other possible explanations for the inconsistent findings include differences in the timing of environmental exposure on the maturing brain [21], or the moderation of environmental influences or developmental effects by genes.

Genetic and environmental influences are not isolated from one another: genes and environment continuously work together throughout brain development [3, 25]. Gene-environment interactions (GxE) could therefore contribute to the heterogeneous findings when genes and environment are studied in isolation. Evidence for GxE effects on brain volumes is scarce and has come primarily from studies of the interaction of adverse life events with functional variants of the *brain-derived neurotrophic factor (BDNF)* gene and the *serotonin-transporter*-linked polymorphic region (*HTTLPR*). These studies showed that carriers of the Met allele of a single nucleotide polymorphism (SNP)—Val66Met—located in *BDNF*, or carriers of the *HTTLPR* short allele, had smaller hippocampal and anterior cingulate cortex (ACC) Gray Matter (GM) volumes when exposed to high levels of (childhood) adversity [26–32]. Although, besides effects on GM volumes in the caudate and several other brain regions for *HTTLPR* short allele carriers, one study also found carriers of two *HTTLPR* long alleles had larger hippocampal and amygdala volumes when exposed to more stressful life events [31]. For *BDNF*, an interaction with early life stress on amygdala GM volume was found as well [26]. Yet, null findings have also been reported in studies of *BDNF* x Childhood adversity effects on hippocampal and amygdala volumes [32, 33]. Importantly, these studies focused only on genetic vulnerability to the effects of adverse life events. Although this is in agreement with the commonly applied diathesis-stress or dual-risk models [34], recent literature also emphasizes the protective effects of positive environments in combination with specific genetic variants [35–38]. Thus, studies reporting on GxE effects in relation to brain volumes so far have revealed only the negative side of the story.

Because the brain develops throughout the lifespan, one must consider developmental brain maturation when studying the effects of genes, the environment, and their interplay on brain volumes. Longitudinal developmental studies have demonstrated that overall cortical and subcortical GM volumes show curvilinear or ‘inverted-U’-like developmental trajectories with age, with the steepest growth found in early childhood and reductions reported from (pre)adolescence onwards, although there is much heterogeneity in developmental trajectories [39–41]. For example, individuals with ADHD appear to have different developmental trajectories of cortical thickness and subcortical volumes when compared to healthy controls [42–44]. It is likely that both direction and degree of environmental effects on neural structures depend on age, as child and adult studies on early adversity often show different or even opposite effects [19]. Moreover, it is likely that the timing of environmental exposures plays a role in which brain regions are sensitive to environmental effects [21]. Together, these findings suggest that developmental differences should be taken into account when studying GxE effects on brain volumes in childhood and adolescence.

In the present cross-sectional study we set out to investigate possible main and interaction effects of candidate genes and the social environment along with the possible moderating role of age on brain volumes in a large sample of children, adolescents, and young adults with and without an ADHD diagnosis. In addition, we investigated whether these effects depended on ADHD severity. We aimed to advance previous studies on brain volumes by investigating positive *and* negative environmental influences. Maternal expressed warmth and criticism as well as positive and deviant peer affiliation were chosen, as both parent and peer influences are important social environments during development and have been associated with neural alterations [22, 45] and shown to affect child externalizing behavior, such as ADHD [46–48]; although the reverse influence (child behavior shaping social environment) has also been shown [49–51]. Furthermore, while these measures are less extreme in comparison to most environmental measures associated with brain structure differences so far (e.g. maltreatment), recent studies have shown evidence for more subtle measures such as parental warmth as well [22, 23]. We focused on candidate variants in the *DAT1*, *5-HTT*, and *DRD4* genes. As reviewed above, these genetic variants have been associated with neural structure volumes, and have been associated with ADHD [52, 53] and shown to interact with the environment in both children with and without ADHD [54]. We focused on total GM, caudate, and putamen volumes, as these have previously been shown to differ between the participants with ADHD and the (healthy) controls of the present sample [43] (i.e. participants overlapped between studies, with the exception that Greven et al. [43] excluded participants with subthreshold ADHD, while the present study excluded participants without information on EE, peer affiliation or genotyping). Individuals with ADHD had smaller total brain and GM volumes compared to controls (2.5–3.0%), whereas for caudate and putamen volumes the differences had a developmental nature; controls showed a decrease in size over age, while individuals with ADHD did not [43]. Because of the importance of developmental effects on brain maturation, we included a large sample with a broad age-range, which allowed us to explore the modulating role of age on the effects of genes, social environment, and their interaction on total GM, caudate, and putamen volumes.

## Materials and Methods

### Participants

Participants were selected from a follow-up (2009–2012) of the Dutch part of the International Multicenter ADHD Genetics (IMAGE) study, performed between 2003–2006 (see [55]). At first enrolment in IMAGE, families with at least one child with combined type ADHD and at least one biological sibling (regardless of ADHD diagnosis) were recruited, in addition to control families with at least one (unaffected) child and no formal or suspected ADHD diagnosis in first-degree family members. Inclusion criteria for children into IMAGE were an age between 5–19 years, European Caucasian descent, IQ ≥ 70, and no diagnosis of autism, epilepsy, general learning difficulties, brain disorders, or known genetic disorders (such as Fragile X syndrome or Down syndrome). All families were reinvited for a follow-up measurement with a mean follow-up period of 5.9 years (*SD* = .74) in Amsterdam or Nijmegen. At this follow-up, a comprehensive assessment protocol was administered, encompassing behavioral questionnaires, a diagnostic interview (assessing ADHD, oppositional defiance disorder [ODD], conduct disorder [CD]), and several neurocognitive measures from all family members, and an extensive MRI scanning protocol in participating children. Participants were asked to withhold use of psychoactive drugs for 48 hours before measurement. To determine ADHD diagnoses at the follow-up measurement, a standardized algorithm was applied to a combination of questionnaires and a semi-structured diagnostic interview. For a detailed description of the assessment protocol, including the diagnostic procedure see [56]. Informed consent was signed by all participants and their parents (for participants under 12 years of age only parents provided consent). The study, including its consent procedure, was approved by the local ethics committees (Centrale Commissie Mensgebonden Onderzoek).

In the current analyses participants were included when information was available on structural MRI, genotype, and maternal expressed emotion (EE) *or* peer affiliation. The final sample included N = 312 participants with ADHD, N = 80 with subthreshold ADHD (i.e., elevated symptoms of ADHD without meeting the full criteria for an ADHD diagnosis), and N = 357 participants without ADHD, from N = 402 families. Sample size depended in particular on the availability of EE and peer affiliation (N = 360 versus N = 726), as EE could only be assessed when the diagnostic interview was administered. This led to an unequal distribution of participants with or without an ADHD diagnosis in the EE (N = 279 with ADHD, N = 45 with subthreshold ADHD, N = 36 without ADHD) versus peer affiliation selection (N = 293 with ADHD, N = 78 with subthreshold ADHD, N = 355 without ADHD). Participant characteristics for the total sample as well as split for high and low ADHD severity are displayed in Table 1.

**Table 1:** Participant characteristics.

	Low ADHD severity (N = 374)			High ADHD severity (N = 368)			Total sample (N = 749)			Group contrast low vs high ADHD severity (*p* < .05)
	*N*	*M*	*SD*	*N*	*M*	*SD*	*N*	*M*	*SD*	
Age	374	16.8	3.5	368	17.3	3.7	749	17.0	3.6	low = high
Family size	317	4.6	0.9	318	4.6	1.0	636	4.6	92.0	low = high
Estimated IQ	370	104.1	13.9	367	98.4	15.4	744	101.2	15.0	low>high
ADHD severity	374	4.9	3.1	368	21.7	8.1	742	13.2	10.4	low<high
Maternal warmth	61	1.7	0.9	294	1.6	0.9	360	1.6	0.9	low = high
Maternal criticism	61	1.5	0.8	294	1.7	0.9	360	1.7	0.9	low<high
Positive PA	370	23.0	3.4	339	21.9	3.6	714	22.5	3.5	low>high
Deviant PA	373	13.6	3.6	348	15.7	4.7	726	14.6	4.3	low<high
Total brain	374	1251.9	120.9	368	1260.7	127.1	749	1256.3	123.8	low = high
Gray matter	374	738.6	72.6	368	738.5	71.6	749	738.6	71.9	low = high
White matter	374	513.3	62.3	368	522.2	68.4	749	517.7	65.5	low<high
Left caudate	374	4.0	0.5	368	4.0	0.5	749	4.0	0.5	low = high
Right caudate	374	4.1	0.5	368	4.1	0.5	749	4.1	0.5	low = high
Total caudate	374	8.1	1.0	368	8.1	0.9	749	8.1	0.9	low = high
Left putamen	374	5.4	0.6	368	5.5	0.6	749	5.4	0.6	low = high
Right putamen	374	5.4	0.6	368	5.5	0.6	749	5.4	0.6	low<high
Total putamen	374	10.8	1.1	368	10.9	1.2	749	10.9	1.2	low<high
ODD diagnosis	11	2.9		93	25.3		104	13.9		low<high
CD diagnosis	0	0.0		20	5.4		20	2.7		low<high
History of stimulant use	32	9.6		252	75.7		289	43.0		low<high
Male	176	47.1		241	65.5		421	56.2		low<high
Collection site (Amsterdam)	219	58.6		158	42.9		380	50.7		low>high
*DAT1*	350			352			709			low = high
9-repeat present	140	40.0		134	38.1		278	39.2		
9-repeat absent^a^	210	60		218	61.9		431	60.8		
*5-HTT*	364			361			732			low = high
Short allele present	321	63.5		241	66.8		478	65.3		
Short allele absent	133	36.5		120	33.2		254	34.7		
*DRD4*	362			360			729			low = high
7-repeat present	136	37.6		125	34.7		264	36.2		
7-repeat absent	226	62.4		235	65.3		465	63.8		

*Note*. Low and high ADHD severity groups based on median split. ODD and CD diagnoses were based on K-SADS structured psychiatric interviews [57]. Estimated IQ was based on two subtests of the WISC/WAIS-III: Vocabulary and Block Design [58, 59].

^a^10/10 genotype.

### Measures

#### Parental expressed emotion

EE was assessed during the semi-structured diagnostic interview, using codings derived from the Camberwell Family Interview [60]. Only ratings of mothers were used in our study, as the data of fathers were far less complete. Warmth was assessed by the tone of voice, spontaneity, sympathy, and/or empathy toward the child (range 0–3). Criticism was assessed by statements which criticized or found fault with the child based on tone of voice and critical phrases (range 0–4) [61, 62]. Adequate inter-rater reliability has been reported for ratings of warmth and criticism using the Camberwell Family Interview (range .78–91 and .79-.86, respectively [63]) and during the first measurement wave (the IMAGE study; range .71–1.00 [64]).

#### Peer affiliation

Peer affiliation was measured with the Friends Inventory [65]. Participants assessed their peers’ behavior on 18 items rated on a 4-point Likert scale (e.g., ‘my friends get good grades’, ‘my friends break the rules’; range 1 = ‘none of my friends are like that’ to 4 = ‘all of my friends are like that’). Scores were summed to yield either a positive or deviant peer affiliation score (each 9 items). Both measures have demonstrated good internal consistency reliability (range .78–92 [48, 66, 67]), and a mean inter-rater reliability of .71 has been reported between teacher and self-reports [67].

#### ADHD severity

ADHD severity was assessed using the DSM-IV total (raw) scores of a parent rating scale (CPRS-R:L; scale N [68]), and either a teaching rating scale (CTRS-R:L; scale N [69]) applied for children <18 years, or a self-report (CAARS-S:S; scale G [70]), applied for children ≥ 18 years. We used the CPRS-R:L and CTRS-R:L or CAARS-S:S as they were assessed in all participants (regardless of diagnostic status). Moreover, using a continuous measure of ADHD severity allowed us to retain as much information as possible, including the variation of scores among unaffected participants.

#### Image acquisition and segmentation

Imaging was conducted at two locations (Amsterdam and Nijmegen) using two similar 1.5 Tesla scanners (Siemens Sonata/ Avanto), the same product 8-channel head-coil, and identical scan protocols. The protocol included two high resolution T1-weighted MP-RAGE anatomical scans (176 sagittal slices, TR = 2730 ms, TE = 2.95 ms, TI = 1000 ms, flip angle = 7 deg, GRAPPA 2, voxel size = 1.0 x 1.0 x 1.0 mm, field of view = 256 mm). MRI scans were manually rated for quality, those that revealed poor quality or motion artefacts (N = 37) were excluded together with scans which yielded relevant incidental findings (N = 18) [71]. Volume estimates were averaged when participants had two good quality scans (N = 741), thereby improving signal-to-noise ratio.

#### Brain volumes

We used volumes that previously have been shown to differ between ADHD and controls included in the present study: of total GM, caudate nucleus, and putamen [43]. The unified procedure of the VBM 8.1 toolbox (http://dbm.neuro.uni-jena.de/vbm/) in SPM (default settings) was used to perform normalization, bias-correction, and segmentation into gray matter, white matter, and cerebrospinal fluid. Total gray and white volumes were calculated by summation of their tissue probability maps. Total brain volume was calculated by summing total gray and white matter volume. For the subcortical volumes, automated FIRST subcortical segmentation was applied to estimate left and right volumes of the caudate and putamen. FIRST, part of FMRIB’s Software Library (FSL), performs registration and shape modeling of the above regions in MNI152 standard space [72].

#### Genotyping

For the IMAGE sample (parents and children), DNA was extracted from blood samples or immortalized cell lines at Rutgers University Cell and DNA Repository, New Jersey, USA. Genetic variants in *DRD4*, *DAT1*, and 5-*HTT* were genotyped by the IMAGE consortium [73, 74]. Standard PCR protocols were used for all VNTR markers and amplified products were visualized on 2% agarose under UV light. Additional NeuroIMAGE samples were collected in the form of a saliva sample using Oragene kits (DNA-Genotek; see www.neuroimage.nl). For those, VNTRs were genotyped using standard PCR protocols at the Department of Human Genetics of the Radboudumc, Nijmegen. After the PCR, fragment length analysis was performed on the ABI prism 3730 Genetic Analyser (Applied Biosystems, Nieuwekerk a/d IJsel, The Netherlands) and results were analyzed with GeneMapper® Software, version 4.0 (Applied Biosystems). No deviations from Hardy-Weinberg Equilibrium were found (*DAT1 p* = .78, 5-*HTT p* = .13, *DRD4 p* = .15). For the data analyses, participants were divided into groups based on the presence or absence of the 9-repeat of the *DAT1* 3’UTR VNTR, the short allele of *HTTLPR*, or the 7-repeat of the *DRD4* exon 3 VNTR, respectively.

### Data Analyses

#### Gene-environment correlations

The presence of gene-environment correlations (rGE) could bias potential GxE by providing an alternative explanation for the relationship between environmental measures and genes [54, 75]. Therefore, Pearson and Spearman correlation analyses were performed to test for rGE between maternal or adolescent genotype and the environmental predictors.

#### Main analyses

All analyses were performed on the total sample, that is, participants with and without ADHD. Linear mixed model analyses investigated the effects of EE, peer affiliation, genotype, and GxE interactions on each volumetric measure (total GM volume and total, left, and right caudate nucleus and putamen volumes). Models were run with and without the interaction term separately. Separate models were run for each environmental predictor: warmth, criticism, and positive and deviant peer affiliation, and for each gene (*DAT1*, *5-HTT*, *DRD4*) as well. Consequently, there were 4 environmental predictors, 3 genes, and 7 outcome measures.

To correct for familial dependency, as a number of participants belonged to the same families, we estimated a random intercept for family in each model. A random intercept accounts for familial dependency by estimating the correlations between cases within families. Age, gender, and collection site were included as covariates. The analyses of the subcortical volumes included total brain volume as an additional covariate. For total GM volume, we added total white matter volume as an extra covariate. Because longitudinal developmental studies have found curvilinear developmental trajectories with age for cortical and subcortical GM volumes, with the steepest growth found in early childhood and reductions reported from (pre)adolescence onwards [39–41], we tested 2- and 3-way interactions with age and age^2^ (i.e., Age/Age^2^xG, Age/Age^2^xE, and Age/Age^2^xGxE) which were dropped from the model when not significant or nominally significant (i.e., did not survive correction for multiple testing). All continuous predictors and covariates were centered around the mean.

#### Multiple testing correction

A multiple comparisons correction was employed which adjusts for correlated tests based on the effective number of independent comparisons (M_eff_) [76]. The M_eff_ was derived from the Eigenvalues of a correlation matrix between the outcome measures adjusted for covariates (age, gender, collection site, and total brain or total white matter volume for subcortical and total GM volumes respectively). In the case of zero correlations between the outcome measures, the M_eff_ adjusted *p*-value would be equivalent to a Bonferroni correction. Thus the M_eff_ procedure is particularly suited for correlated comparisons (such as total, left and right putamen volumes) and corrects for multiple testing balancing between being overly lenient or conservative. The effective number of comparisons was determined to be 3.55 and the adjusted *p*-value threshold: 0.05/3.55 = .014.

#### Sensitivity analyses

Sensitivity analyses were performed when significant effects that survived the multiple correction threshold were found. First, Regions of significance (RoS), simple slope, and slope difference tests were performed with an application designed for probing 2- and 3-way interactions (http://www.jeremydawson.co.uk/slopes.htm [77]). For interactions with age^2^, slope tests were estimated with non-quadratic age. Second, to investigate the role of ADHD severity, analyses were rerun including main and interaction effects of ADHD severity. Furthermore, separate sensitivity analyses were performed to check whether significant effects were present in participants while sequentially controlling for effects of medication history, estimated IQ, and comorbid ODD or CD diagnosis. All analyses (except for RoS and slope tests) were performed with the Statistical Package for the Social Sciences, version 20.0.

## Results

Analyses of gene-environment correlations (rGEs) revealed significant correlations between maternal warmth and adolescent *DAT1* (*r* = -.11, *p* = .045), *5-HTT* (*r* = -.11, *p* = .040), and *DRD4* genotypes (*r* = -.11, *p* = .048), and maternal *DAT1* genotypes (*r* = -.15, *p* = .005). We also found a significant correlation between maternal criticism and adolescent *DAT1* genotypes (*r* = .12, *p* = .024) (see S1 Table). Considering the size of these associations, there was no reason to believe that these rGEs may have biased significant GxE interactions. In the next sections on main and GxE effects, only results of the mixed model analyses that survived correction for multiple testing are discussed (*p* < .014). Nominal significant results can be found in the Supporting Information (SI) in S2 and S3 Tables.

### Main effects of candidate genes and environments and the effect of age

No significant main effects of maternal warmth or criticism were found (all *p*-values >.166; see S2 Table). A small main effect of deviant peer affiliation was found on the left caudate volume (*B* = -.01, *p* = .012), indicating that more deviant peer affiliation was related to smaller caudate volumes. This effect was also present in the total and right caudate volumes, but did not survive correction for multiple testing (*B* = -.02, *p* = .017; *B* = -.01, *p* = .031 respectively). No main gene effects were found that survived the correction for multiple testing (all *p*-values >.015).

**Fig 1:**
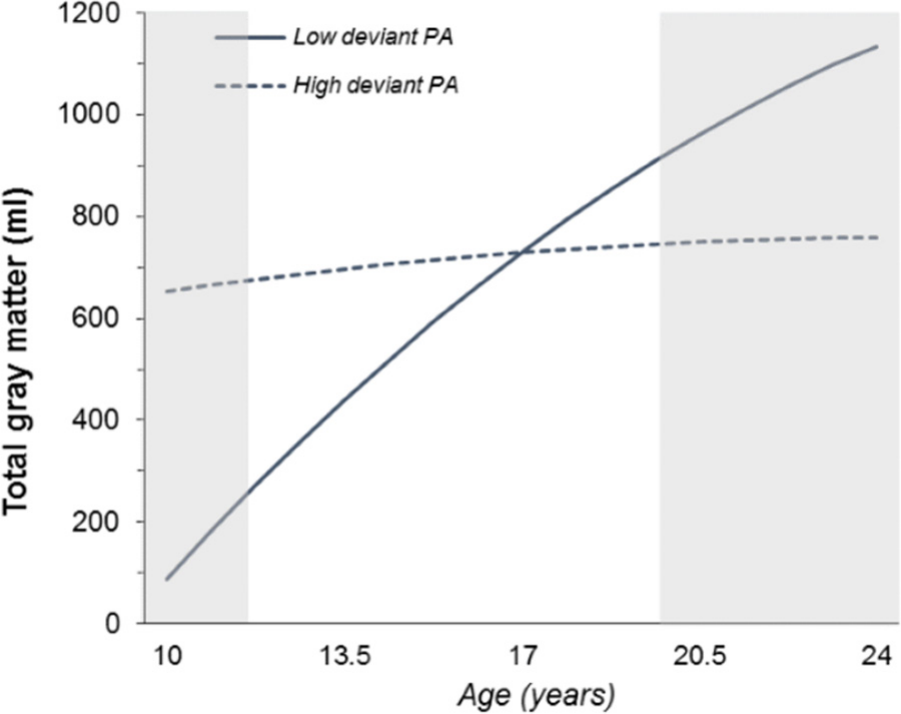
Two-way interaction between deviant peer affiliation (low: - 2SD; high: + 2SD) and age^2^ on total gray matter volume. PA = peer affiliation. Regression lines show the predicted values. The shaded areas indicate the regions of significance (RoS), lower threshold: X = 10.44 years; upper threshold: X = 20.16 years.

Investigation of the effects of linear and non-linear age yielded a significant interaction between deviant peer affiliation and the quadratic effects of age (age^2^) on total GM volume (*p =* .001). As shown in Fig 1, participants with low deviant peer affiliations (- 2SD) had larger total GM volumes when older (*p* = .001), while participants with high deviant peer affiliations (+ 2SD) showed no association with age (*p* = .112).

### GxE interactions and the role of age

**Fig 2:**
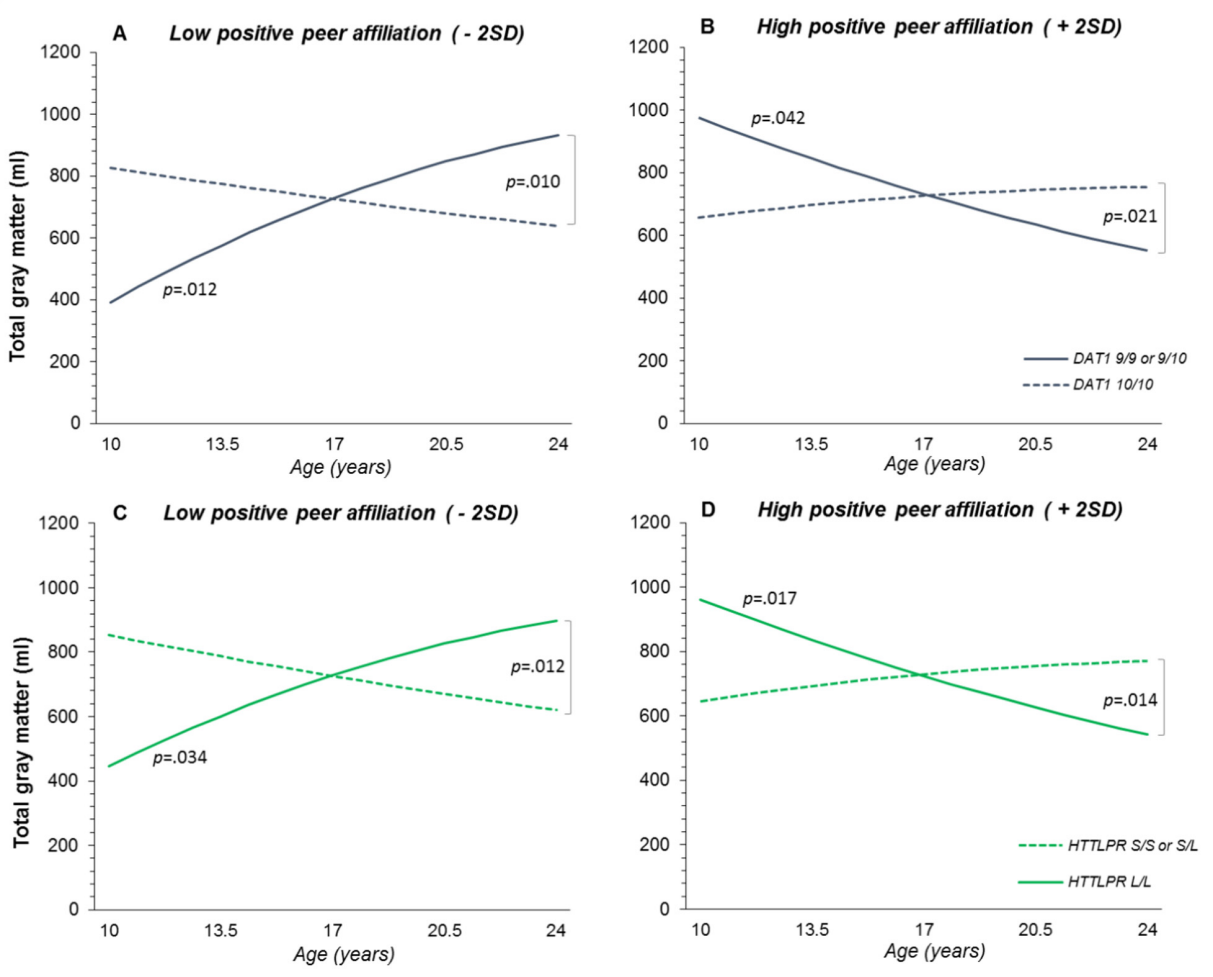
Three-way interactions of *DAT1* and *5-HTT* with positive peer affiliation and age^2^ on total brain volume. (A & B) Three-way interaction between *DAT1*, positive peer affiliation, and age^2^ on total gray matter volume, shown separately for low (A; - 2SD) and high positive peer affiliation (B; + 2SD). (C & D) Three-way interaction between *5-HTT*, positive peer affiliation, and age^2^ on total gray matter volume, shown separately for low (C; - 2SD) and high positive peer affiliation (D; + 2SD). Regression lines show predicted values. *P*-values indicate significant slopes and significant slope differences.

For total GM volume we found two 3-way interactions between positive peer affiliation, *DAT1*, and age^2^ (*p* = .007), as well as between positive peer affiliation, *5-HTT*, and age^2^ (*p* = .012). Simple slope analyses revealed significant slopes (i.e., different from slope = 0) for carriers of the *DAT1* 9-repeat or two *5-HTT* long alleles when scoring either low or high on positive peer affiliation (- 2SD: *p*_*DAT1*_ = .012, *p*_*5-HTT*_ = .034; + 2SD: *p*_*DAT1*_ = .042, *p*_*5-HTT*_ = .017). These slopes differed significantly from each other as well (*p*_*DAT1*_ = .009, *p*_*5-HTT*_ = .014). As shown in Fig 2, carriers of the *DAT1* 9-repeat or two *5-HTT* long alleles with low positive peer affiliations had larger GM volumes with age, while participants with the same genotype, but high positive peer affiliations had smaller GM volumes with age. Slopes for participants with the *DAT1* 10/10 genotype or *5-HTT* short allele were not significant (- 2SD: *p*_*DAT1*_ = .368, *p*_*5-HTT*_ = .153; + 2SD: *p*_*DAT1*_ = .272, *p*_*5-HTT*_ = .346). Still, slopes of *DAT1* 9-repeat carriers and 10-repeat homozygotes differed significantly when they scored low (- 2SD, *p* = .010) or high on positive peer affiliation (+ 2SD, *p* = .021). The same was true for slopes of *5-HTT* short allele carriers and long allele homozygotes (- 2SD, *p* = .012; + 2SD, *p* = .014).

**Fig 3:**
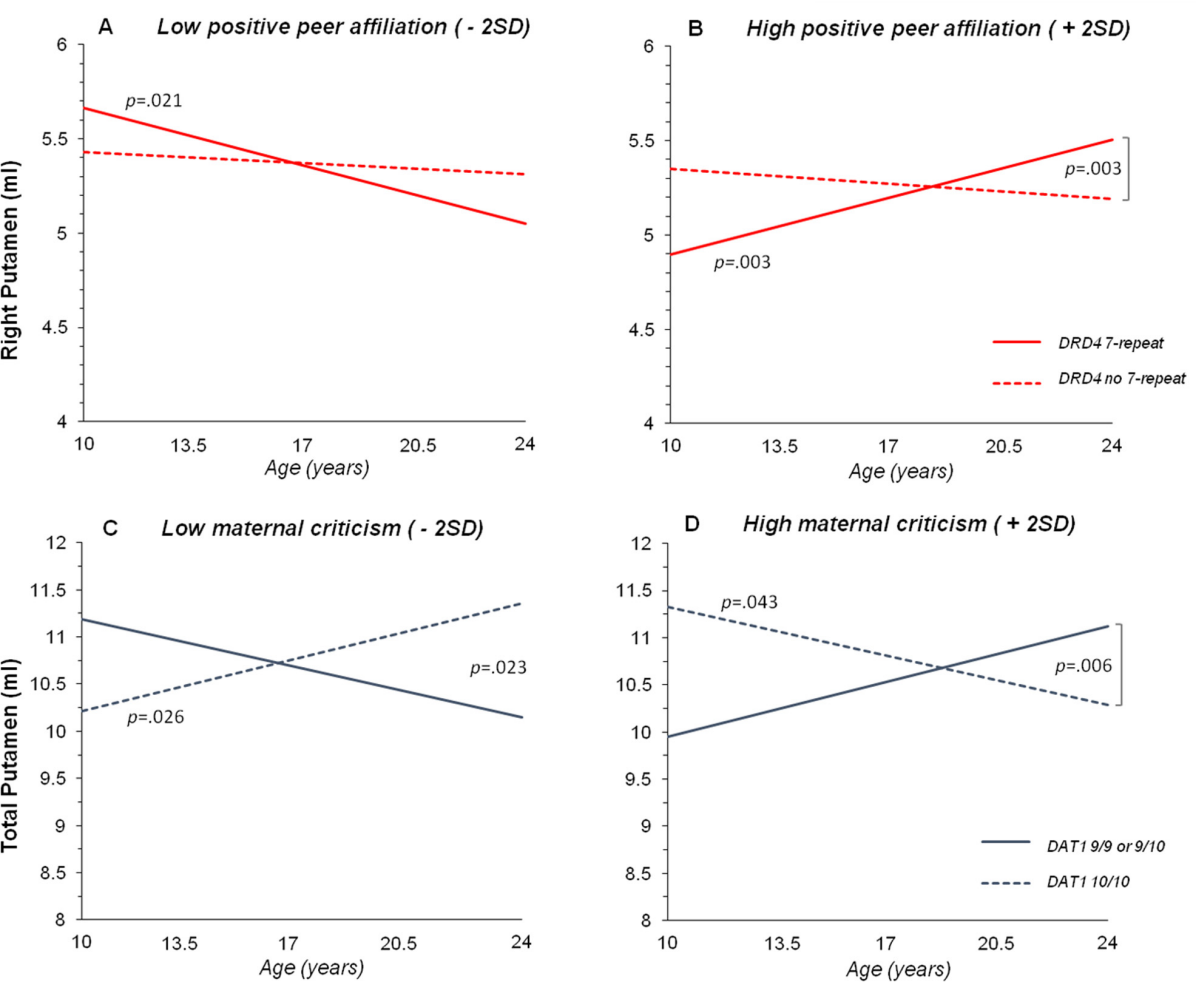
Three-way interactions of *DRD4*, positive peer affiliation and age, and *DAT1*, maternal criticism and age on putamen volume. (A & B) Three-way interaction between *DRD4*, positive peer affiliation, and age on right putamen volume, shown separately for low (A; - 2SD) and high positive peer affiliation (B; + 2SD). (C & D) Three-way interaction between *DAT1*, maternal criticism, and age on total putamen volume, shown separately for low (C; - 2SD) and high maternal criticism (D; + 2SD). Regression lines show predicted values. *P*-values indicate significant slopes and significant slope differences.

For putamen volume significant 3-way interactions with age were found as well. Fig 3A and 3B show the interaction between *DRD4*, positive peer affiliation, and age on the right putamen volume (*p* = .012). Significant slopes were found for *DRD4* 7-repeat carriers scoring either low or high on positive peer affiliation (- 2SD: *p* = .021; + 2SD: *p* = .003), and the difference between the two slopes was significant as well (*p* = .004). Thus, 7-repeat carriers showed differential associations between the right putamen volume and age depending on the amount of positive peer affiliation, i.e., a negative association when scoring low on positive peer affiliation, but positive when scoring high on positive peer affiliation. Although slopes of participants without the *DRD4* 7-repeat allele were not significant (- 2SD: *p* = .492; + 2SD: *p* = .308), significant slope differences were found between 7-repeat carriers scoring high on positive peer affiliation and participants without the 7-repeat with either low (*p* = .007) or high positive peer affiliations (*p* = .003).

Finally, an interaction was found between *DAT1*, criticism, and age on total putamen volume (*p* = .005). This effect was present in both left and right putamen volumes (left: *p* = .009; right: *p* = .006). Here, significant slopes were found for the *DAT1* 10/10 genotype with either low or high maternal criticism (- 2SD: *p* = .026; + 2SD: *p* = .043), which differed significantly from each other as well (*p* = .016). As can be seen in Fig 3C and 3D, participants with two 10-repeat alleles exposed to high maternal criticism had smaller putamen volumes with age, but participants exposed to low criticism had larger putamen volumes with age. Slopes of *DAT1* 9-repeat carriers were not significant (- 2SD years: *p* = .095; + 2SD years: *p* = .155). Analyses of slope differences revealed significant differences between *DAT1* 9-repeat carriers and 10-repeat homozygotes when scoring low (- 2SD, *p* = .023) or high on maternal criticism (- 2SD, *p* = .006). Highly similar results were found when investigating left and right putamen volumes separately.

### Sensitivity analyses

Through sensitivity analyses we investigated the possible role of ADHD on the significant main effects and interactions described above. When the analyses were rerun including main and interaction effects of ADHD severity, no significant interactions with ADHD severity were found (all *p*-values >.133). Including a main effect of ADHD severity did not change the aforementioned significant main or GxExAge/Age^2^ effects either. The *p*-value of the interaction between *DAT1*, positive peer affiliation, and age^2^ did drop slightly, thereby becoming nominally significant (*p* = .018). The same was true for the interaction between *5-HTT*, positive peer affiliation, and age^2^ (*p* = .015). Furthermore, rerunning analyses consecutively including IQ, ODD, CD, or medication history in the model yielded highly similar results. Note that for the main effect of deviant peer affiliation, the *p*-value dropped to nominal significance (all *p*-values < .037) when including IQ, ODD, or CD. Similarly, *p*-values dropped slightly when ODD or medication history was included for the interaction between *5-HTT*, positive peer affiliation, and age^2^ on total GM volume and the 3-way interactions on putamen volume (all *p*-values < .043).

## Discussion

We investigated the effects of functional variants in the *5-HTT*, *DAT1*, and *DRD4* genes, the social environment, and the interactions between genes and environment on brain volumes in a large sample of children, adolescents, and young adults with and without ADHD. We took a developmental approach, examining the impact of age on main and interaction effects of candidate genes and social environments. As expected, few (i.e., one) main effect was observed, of deviant peer affiliation on left caudate volume. Instead, we observed a complex pattern of the following two-way and three-way interactions: an interaction between deviant peer affiliation and age^2^ for total GM volume, and between *5-HTT*, *DAT1*, or *DRD4* variants and positive peer affiliation or maternal criticism on total GM and putamen volumes. These findings were independent of ADHD severity. The results extend findings from twin studies [78–80] that genetic effects are developmentally sensitive by showing gene-by-environment interactions appear to be developmentally sensitive as well.

We found different age-effects for total GM and putamen volumes, depending on genotype and/or environmental exposure. In agreement with age-related reductions of total GM volume found in longitudinal studies [39, 40], participants scoring high on positive peer affiliation carrying the *DAT1* 9-repeat or two *HTTLPR* long alleles had smaller total GM volumes with age. Moreover, participants with the same genotype, but *low* positive peer affiliation had *larger* GM volumes with age. These findings are in line with a longitudinal study reporting regional GM reductions with age in adolescents exposed to high positive maternal behavior, but increased putamen volumes when exposed to maternal aggression [22]. However, we also found positive associations between total GM and age in participants scoring low on deviant peer affiliation, regardless of genotype, while participants with high deviant peer affiliation had no association with age. Similarly, for putamen volume, carriers of the *DRD4* 7-repeat or *DAT1* 10/10 genotypes had larger volumes over age when exposed to high positive peer affiliation or low maternal criticism respectively, but opposite patterns, i.e., smaller volumes over age, when exposed to low positive peer affiliation or high criticism.

Although, equivalent to total GM, decreased putamen volumes over age have been reported [40], different age-effects of putamen volume have been found for participants with ADHD in comparison to healthy controls by our group [43]. Thus, one might expect the opposing age patterns found in the present study to be related to ADHD. However, none of the current findings were moderated by ADHD severity, suggesting they contribute to total GM, putamen, and caudate volumes in a more general manner, similar for individuals with and without ADHD.

Previous GxE studies on brain volumes have been few and have mostly included (young) adults, leaving GxE effects on child or adolescent brain volumes under-investigated. So far, of the included candidate genes, only the *5-HTT* gene has previously been reported to interact with stressful life events [29–31, 81]. In a previous study using the present sample, stress exposure was associated with less GM volume in the precental gyrus, middle and superior frontal gyrus, frontal pole, and cingulate gyrus in carriers of the *5-HTT* short allele compared to long allele homozygotes [81]. Similar results have been found in adults studies, showing smaller hippocampal volumes for carriers of the *HTTLPR* short allele when exposed to childhood adversity [29, 30], though Canli et al. [31] reported that only long allele homozygotes had a positive association between stressful life events and hippocampal or amygdala GM volumes. In other regions, such as the ACC or caudate, both short and long allele carriers showed opposite associations with life stress. These opposite effects are in line with the differential effects of *DAT1* variants we found on total GM versus putamen volumes. That is, *DAT1* 9-repeat carriers showed age-dependent associations between positive peer affiliation and GM volumes, while *DAT1* 10-repeat homozygotes showed differential associations between maternal criticism and putamen volumes. This could suggest that different variants of the same gene are susceptible to different environments, which could further depend on which brain region is focused on. In contrast to what we expected, both genotypes showed the same direction of association with two opposing environments; participants with high positive peer affiliations or high maternal criticism both had smaller GM volumes with age, while participants with low positive peer affiliations or low criticism showed the opposite pattern.

Besides different effects of the same gene, differential effects of positive peer affiliation were found for carriers of specific gene variants as well, i.e., participants scoring low on positive peer affiliation with the *DAT1* 9-repeat or two *HTTLPR* long alleles had larger total GM volume, while those with low positive peer affiliation and the *DRD4* 7-repeat allele had smaller putamen volumes with age, with the opposite pattern found in participants scoring high on positive peer affiliation. This suggests positive peer affiliation can have different effects depending on which brain volume or genotype is focused on. What our findings most consistently show is that (for carriers of specific gene variants) the direction of associations between certain social environments and brain volumes depends on developmental stage. This is illustrated by our finding that, for carriers of the same gene variants, low positive peer affiliation or high maternal criticism was related to smaller total GM or putamen volumes in preadolescents, but to larger volumes in young adults. Such differential effects were found in each of the reported two- and three-way interactions. In agreement, our group has shown that associations between the *5-HTT*, *DAT1*, *DRD4* and neurocognitive functioning, such as inhibition and working memory, depended on age as well [82]. These findings highlight the importance of including age when studying genetic and environmental effects on the neural architecture of children, adolescents and young adults, as the direction of associations likely depend on developmental stage.

There are several possible explanations for the finding of developmentally sensitive GxE effects on total GM and putamen volumes. Neurotransmitters have an important role in synaptic and neural plasticity [83], and the age-dependent GxE effects may thus be related to differences in dopamine and serotonin availability associated with *DRD4*, *DAT1* and *5-HTT* variants. Specifically, the *DRD4* 7-repeat and *HTTLPR* short alleles are associated with decreased transcriptional activity, leading to increased levels of dopamine and serotonin [84, 85]. For *DAT1* there are mixed results about whether the 9- or 10-repeat shows increased or decreased expression. However, a recent meta-analysis showed that the 9-repeat allele was associated with increased *in vivo* striatal dopamine transporter activity in adults independent of the presence of neuropsychiatric disorders [86]. Expression levels of genes can differ across developmental stages [87] and dopamine and serotonin levels in the brain have been shown to differ over age as well [88]. Besides genetic and developmental influences, environmental factors, such as maternal deprivation or environmental enrichment, have also been associated with differential neurotransmitter levels [89]. Furthermore, other processes during brain development, including neuronal pruning, myelination and hormonal influences are believed to be influenced by genetic and environmental factors as well [24, 90]. Together, these findings reveal many important developmental processes, steered by environmental and genetic factors, which together determine one’s neural architecture.

For caudate volume, one developmentally stable main effect was found. Higher deviant peer affiliation was associated with slightly smaller caudate volumes. This agrees with a previous study that found early life stress was related to smaller caudate volumes in adults [91]. Similarly, smaller volumes of other brain regions, such as the hippocampus, have been associated with adverse psychosocial experiences [18, 19, 21], but findings are not consistent since null-findings, and even larger volumes in relation to adverse environments have been reported [18, 19, 21].

All but one of the reported effects in the current study were found in relation to peer affiliation. While peer influences have been linked to functional brain differences [45, 92] and white matter structure [93], no prior studies have addressed associations with volumetric alterations. Besides peer presence and peer verbal abuse investigated in the aforementioned studies [45, 92, 93], our results indicate that the type of peers seems to be relevant for structural brain differences. The associations between deviant or positive peer affiliation and brain volume alterations could be the result of intrinsic or extrinsic factors not investigated in this study. An example would be that structural brain alterations are first and foremost associated with intrinsic personality traits (such as high sensation seeking or low conscientiousness) that fit well with deviant peer affiliation. Indeed, studies have revealed both positive and negative associations of traits such as extraversion and conscientiousness with (regional) gray and white matter volumes [94–97]. Likewise, circumstances such as neighborhood quality could be an example of extrinsic factors underlying peer affiliation. Future studies are needed to investigate whether the association between peer affiliation and brain volumes reflects an effect of other factors such as personality traits or environmental conditions.

Our findings should be viewed in the light of certain strengths and limitations. Strengths were the use of a large well-characterized sample, inclusion of both positive and negative environments, assessment of both parental- and peer influences, and a developmentally sensitive approach. A limitation has been the cross-sectional MRI study design, only longitudinal MRI studies can clarify the direction of causality. Establishing the direction of effects is particularly difficult when focusing on the social environment as it can also (partly) be driven by child effects. Indeed, both maternal EE and peer affiliations have not only been suggested to influence child behavior, but in turn be influenced by child behavior as well [46–49, 98–101]. Thus, it is most likely that bidirectional effects exist between the social environment and child behavior. Furthermore, not all participants included had an expressed emotion (EE) measurement, as the design of our study was such that EE was only assessed when a full diagnostic interview was administered. This led to loss of power, and unequal numbers and an unequal distribution of ADHD and controls in the EE versus peer affiliation analyses. Nevertheless, sensitivity analyses revealed no significant effects of ADHD severity on the reported findings. In addition, although included measures were chosen a-priori based on previous literature, our findings only shed light on a small part of brain variability based on only a few gene variants. Finally, considering the novel and explorative nature of our study, we did not employ a very stringent correction for multiple testing. If we would have applied the most stringent correction—correcting for the total number of environmental measures (n = 4), genes (n = 3), and outcome measures—the corrected *p*-threshold would have been *p =* 0.05/(4*3*3.55) = .0012 instead of *p* = .014. In this case, all but the interaction between deviant peer affiliation and age would not have survived the correction for multiple testing. Therefore, we emphasize the necessity of independent replication studies.

In conclusion, beside a main effect of deviant peer affiliation on caudate volume, we found multiple developmentally sensitive GxE effects on total GM and putamen volume. Despite previously reported differences in total GM, caudate, and putamen volumes between individuals with ADHD and healthy controls, our results were independent of ADHD severity. Both children, adolescents, and young adults with and without ADHD showed differential sensitivity to environmental influences, depending on genotype and age. This suggests that interactions between genes and the social environment contribute in a general way to the included cortical and subcortical brain volumes and are not specific for ADHD. Nevertheless, variation in these brain volume sizes could be relevant for other clinical or functional outcomes not studied here. The caudate nucleus and putamen, for example, have been linked to cognitive, executive and emotional functioning [102].

While it is clear that our complex findings are in need of replication, our results stress the importance of a developmentally sensitive approach when investigating genetic and social environmental influences on interindividual brain volume variability. The failure to do so could potentially explain the diverse and sometimes opposing results of main environmental and genetic effects on brain volumes reported so far.

## Supporting Information

S1 Table Correlation analyses between environmental measures, maternal or adolescent’s genotype, brain volumes and ADHD severity. Pearson correlation analyses were performed for all variables, except for correlations with peer affiliation and ADHD severity, for which Spearman correlations analyses were performed. ^a^ 9-repeat allele present or absent; ^b^ short allele present or absent; ^c^ 7-repeat allele present or absent short allele present or absent. * Significant at *p* ≤ .05 ** Significant at *p* ≤ .01
(DOCX)

S2 Table Mixed model analyses testing the separate main effects of maternal expressed emotion, peer affiliation and plasticity genes on brain volumes. EE = expressed emotion; PA = peer affiliation. ^a^ Reference group: 10/10 absent; ^b^ reference group: short allele absent; ^c^ reference group: 7-repeat absent. Findings in bold are significant after correction for multiple testing (*p* < .014), findings in bold and italic are nominally significant (i.e. not significant after correction for multiple testing; *p* ≤ .05). All analyses were corrected for age, gender, and collection site. Additionally, total brain volume was included in analyses of subcortical volumes, and total white matter in analyses of total gray matter as well.
(DOCX)

S3 Table Mixed model analyses testing interaction effects between plasticity genes and maternal expressed emotion or peer affiliation on brain volumes. PA = peer affiliation. ^a^ Reference group: 10/10 absent; ^b^ reference group: short allele absent; ^c^ reference group: 7-repeat absent. Findings in bold are significant after correction for multiple testing (*p* < .014), findings in bold and italic are nominally significant (i.e., not significant after correction for multiple testing; *p* ≤ .05). All analyses were corrected for age, gender, and collection site. Additionally, total brain volume was included in analyses of subcortical volumes, and total white matter in analyses of total gray matter as well. In each model 2- and 3-way interactions with age or age^2^ were tested and removed when not significant (*p* > .05) or nominally significant (*p* ≥ .014).
(DOCX)

## References

[1] Frodl T, Skokauskas N. Meta-analysis of structural MRI studies in children and adults with attention deficit hyperactivity disorder indicates treatment effects. Acta psychiatrica Scandinavica. 2012;125(2):114–26. Epub 2011/11/29. 10.1111/j.1600-0447.2011.01786.x . 22118249

[2] Goodkind M, Eickhoff SB, Oathes DJ, Jiang Y, Chang A, Jones-Hagata LB, et al Identification of a common neurobiological substrate for mental illness. JAMA psychiatry. 2015;72(4):305–15. Epub 2015/02/05. 10.1001/jamapsychiatry.2014.2206 . 25651064 PMC4791058

[3] Gu J, Kanai R. What contributes to individual differences in brain structure? Frontiers in human neuroscience. 2014;8:262 Epub 2014/05/09. 10.3389/fnhum.2014.00262 24808848 PMC4009419

[4] Peper JS, Brouwer RM, Boomsma DI, Kahn RS, Hulshoff Pol HE. Genetic influences on human brain structure: a review of brain imaging studies in twins. Human brain mapping. 2007;28(6):464–73. Epub 2007/04/07. 10.1002/hbm.20398 . 17415783 PMC6871295

[5] den Braber A, Bohlken MM, Brouwer RM, van 't Ent D, Kanai R, Kahn RS, et al Heritability of subcortical brain measures: a perspective for future genome-wide association studies. NeuroImage. 2013;83:98–102. Epub 2013/06/19. 10.1016/j.neuroimage.2013.06.027 . 23770413

[6] Stein JL, Hibar DP, Madsen SK, Khamis M, McMahon KL, de Zubicaray GI, et al Discovery and replication of dopamine-related gene effects on caudate volume in young and elderly populations (N = 1198) using genome-wide search. Molecular psychiatry. 2011;16(9):927–37, 881. Epub 2011/04/20. 10.1038/mp.2011.32 21502949 PMC3140560

[7] Stein JL, Medland SE, Vasquez AA, Hibar DP, Senstad RE, Winkler AM, et al Identification of common variants associated with human hippocampal and intracranial volumes. Nature genetics. 2012;44(5):552–61. Epub 2012/04/17. 10.1038/ng.2250 22504417 PMC3635491

[8] Hibar DP, Stein JL, Renteria ME, Arias-Vasquez A, Desrivieres S, Jahanshad N, et al Common genetic variants influence human subcortical brain structures. Nature. 2015;520(7546):224–9. Epub 2015/01/22. 10.1038/nature14101 25607358 PMC4393366

[9] Frodl T, Koutsouleris N, Bottlender R, Born C, Jager M, Morgenthaler M, et al Reduced gray matter brain volumes are associated with variants of the serotonin transporter gene in major depression. Molecular psychiatry. 2008;13(12):1093–101. Epub 2008/11/15. 10.1038/mp.2008.62 . 19008895

[10] Atmaca M, Onalan E, Yildirim H, Yuce H, Koc M, Korkmaz S, et al Serotonin transporter gene polymorphism implicates reduced orbito-frontal cortex in obsessive-compulsive disorder. Journal of anxiety disorders. 2011;25(5):680–5. Epub 2011/03/29. 10.1016/j.janxdis.2011.03.002 . 21441009

[11] Hickie IB, Naismith SL, Ward PB, Scott EM, Mitchell PB, Schofield PR, et al Serotonin transporter gene status predicts caudate nucleus but not amygdala or hippocampal volumes in older persons with major depression. Journal of affective disorders. 2007;98(1–2):137–42. Epub 2006/08/26. 10.1016/j.jad.2006.07.010 . 16930719

[12] Taylor WD, Steffens DC, Payne ME, MacFall JR, Marchuk DA, Svenson IK, et al Influence of serotonin transporter promoter region polymorphisms on hippocampal volumes in late-life depression. Arch Gen Psychiatry. 2005;62(5):537–44. Epub 2005/05/04. 10.1001/archpsyc.62.5.537 . 15867107

[13] Price JS, Strong J, Eliassen J, McQueeny T, Miller M, Padula CB, et al Serotonin transporter gene moderates associations between mood, memory and hippocampal volume. Behav Brain Res. 2013;242:158–65. Epub 2012/12/26. 10.1016/j.bbr.2012.11.013 . 23266326 PMC4433017

[14] Durston S, Fossella JA, Casey BJ, Hulshoff Pol HE, Galvan A, Schnack HG, et al Differential effects of DRD4 and DAT1 genotype on fronto-striatal gray matter volumes in a sample of subjects with attention deficit hyperactivity disorder, their unaffected siblings, and controls. Molecular psychiatry. 2005;10(7):678–85. Epub 2005/02/23. 10.1038/sj.mp.4001649 . 15724142

[15] Shook D, Brady C, Lee PS, Kenealy L, Murphy ER, Gaillard WD, et al Effect of dopamine transporter genotype on caudate volume in childhood ADHD and controls. American journal of medical genetics Part B, Neuropsychiatric genetics: the official publication of the International Society of Psychiatric Genetics. 2011;156B(1):28–35. Epub 2010/10/20. 10.1002/ajmg.b.31132 20957668 PMC3010298

[16] Shaw P, Gornick M, Lerch J, Addington A, Seal J, Greenstein D, et al Polymorphisms of the dopamine D4 receptor, clinical outcome, and cortical structure in attention-deficit/hyperactivity disorder. Arch Gen Psychiatry. 2007;64(8):921–31. Epub 2007/08/08. 10.1001/archpsyc.64.8.921 . 17679637

[17] Teicher MH, Tomoda A, Andersen SL. Neurobiological consequences of early stress and childhood maltreatment: are results from human and animal studies comparable? Annals of the New York Academy of Sciences. 2006;1071:313–23. Epub 2006/08/08. 10.1196/annals.1364.024 . 16891580

[18] Belsky J, de Haan M. Annual Research Review: Parenting and children's brain development: the end of the beginning. Journal of Child Psychology and Psychiatry. 2011;52(4):409–28. Epub 2010/07/16. 10.1111/j.1469-7610.2010.02281.x . 20626527

[19] Tottenham N, Sheridan MA. A review of adversity, the amygdala and the hippocampus: a consideration of developmental timing. Frontiers in human neuroscience. 2009;3:68 Epub 2010/02/18. 10.3389/neuro.09.068.2009 20161700 PMC2813726

[20] Hart H, Rubia K. Neuroimaging of child abuse: a critical review. Frontiers in human neuroscience. 2012;6:52 Epub 2012/03/30. 10.3389/fnhum.2012.00052 22457645 PMC3307045

[21] Teicher MH, Samson JA. Childhood maltreatment and psychopathology: A case for ecophenotypic variants as clinically and neurobiologically distinct subtypes. American journal of psychiatry. 2013;170(10):1114–33. Epub 2013/08/29. 10.1176/appi.ajp.2013.12070957 23982148 PMC3928064

[22] Whittle S, Simmons JG, Dennison M, Vijayakumar N, Schwartz O, Yap MB, et al Positive parenting predicts the development of adolescent brain structure: a longitudinal study. Developmental cognitive neuroscience. 2014;8:7–17. Epub 2013/11/26. 10.1016/j.dcn.2013.10.006 . 24269113 PMC6990097

[23] Luby JL, Barch DM, Belden A, Gaffrey MS, Tillman R, Babb C, et al Maternal support in early childhood predicts larger hippocampal volumes at school age. Proceedings of the National Academy of Sciences of the United States of America. 2012;109(8):2854–9. Epub 2012/02/07. 10.1073/pnas.1118003109 22308421 PMC3286943

[24] Sale A, Berardi N, Maffei L. Environment and brain plasticity: towards an endogenous pharmacotherapy. Physiological reviews. 2014;94(1):189–234. Epub 2014/01/03. 10.1152/physrev.00036.2012 . 24382886

[25] Hyde LW, Bogdan R, Hariri AR. Understanding risk for psychopathology through imaging gene-environment interactions. Trends in cognitive sciences. 2011;15(9):417–27. Epub 2011/08/16. 10.1016/j.tics.2011.07.001 21839667 PMC3163727

[26] Gatt JM, Nemeroff CB, Dobson-Stone C, Paul RH, Bryant RA, Schofield PR, et al Interactions between BDNF Val66Met polymorphism and early life stress predict brain and arousal pathways to syndromal depression and anxiety. Molecular psychiatry. 2009;14(7):681–95. Epub 2009/01/21. 10.1038/mp.2008.143 . 19153574

[27] Carballedo A, Morris D, Zill P, Fahey C, Reinhold E, Meisenzahl E, et al Brain-derived neurotrophic factor Val66Met polymorphism and early life adversity affect hippocampal volume. American journal of medical genetics Part B, Neuropsychiatric genetics: the official publication of the International Society of Psychiatric Genetics. 2013;162B(2):183–90. Epub 2013/01/24. 10.1002/ajmg.b.32130 . 23341118

[28] Rabl U, Meyer BM, Diers K, Bartova L, Berger A, Mandorfer D, et al Additive gene-environment effects on hippocampal structure in healthy humans. The Journal of neuroscience: the official journal of the Society for Neuroscience. 2014;34(30):9917–26. Epub 2014/07/25. 10.1523/JNEUROSCI.3113-13.2014 . 25057194 PMC4107408

[29] Everaerd D, Gerritsen L, Rijpkema M, Frodl T, van Oostrom I, Franke B, et al Sex modulates the interactive effect of the serotonin transporter gene polymorphism and childhood adversity on hippocampal volume. Neuropsychopharmacology. 2012;37(8):1848–55. Epub 2012/03/22. 10.1038/npp.2012.32 22434222 PMC3376317

[30] Frodl T, Reinhold E, Koutsouleris N, Donohoe G, Bondy B, Reiser M, et al Childhood stress, serotonin transporter gene and brain structures in major depression. Neuropsychopharmacology. 2010;35(6):1383–90. Epub 2010/02/12. 10.1038/npp.2010.8 20147891 PMC3055341

[31] Canli T, Qiu M, Omura K, Congdon E, Haas BW, Amin Z, et al Neural correlates of epigenesis. Proceedings of the National Academy of Sciences of the United States of America. 2006;103(43):16033–8. Epub 2006/10/13. 10.1073/pnas.0601674103 17032778 PMC1592642

[32] Gerritsen L, Tendolkar I, Franke B, Vasquez AA, Kooijman S, Buitelaar J, et al BDNF Val66Met genotype modulates the effect of childhood adversity on subgenual anterior cingulate cortex volume in healthy subjects. Molecular psychiatry. 2012;17(6):597–603. Epub 2011/05/18. 10.1038/mp.2011.51 . 21577214

[33] Hernaus D, van Winkel R, Gronenschild E, Habets P, Kenis G, Marcelis M, et al Brain-derived neurotrophic factor/FK506-binding protein 5 genotype by childhood trauma interactions do not impact on hippocampal volume and cognitive performance. PloS one. 2014;9(3):e92722 Epub 2014/03/25. 10.1371/journal.pone.0092722 24658422 PMC3962453

[34] Zubin J, Spring B. Vulnerability—a new view of schizophrenia. Journal of abnormal psychology. 1977;86(2):103–26. Epub 1977/04/01. . 858828 10.1037//0021-843x.86.2.103

[35] Belsky J, Jonassaint C, Pluess M, Stanton M, Brummett B, Williams R. Vulnerability genes or plasticity genes? Molecular psychiatry. 2009;14(8):746–54. Epub 2009/05/21. 10.1038/mp.2009.44 19455150 PMC2834322

[36] Belsky J. Variation in Susceptibility to Environmental Influence: An Evolutionary Argument. Psychological Inquiry. 1997;8(3):182–6. 10.1207/s15327965pli0803_3 .

[37] Belsky J. Differential susceptibility to rearing influences: An evolutionary hypothesis and some evidence In: Ellis BJ, Bjorklund DF, editors. Origins of the social mind: Evolutionary psychology and child development. New York: Guilford Press; 2005 p. 139–63.

[38] Ellis BJ, Boyce WT, Belsky J, Bakermans-Kranenburg MJ, van IJzendoorn MH. Differential susceptibility to the environment: an evolutionary-neurodevelopmental theory. Development and psychopathology. 2011;23(1):7–28. Epub 2011/01/26. 10.1017/S0954579410000611 . 21262036

[39] Raznahan A, Shaw PW, Lerch JP, Clasen LS, Greenstein D, Berman R, et al Longitudinal four-dimensional mapping of subcortical anatomy in human development. Proceedings of the National Academy of Sciences of the United States of America. 2014;111(4):1592–7. Epub 2014/01/30. 10.1073/pnas.1316911111 24474784 PMC3910572

[40] Brain Development Cooperative G. Total and regional brain volumes in a population-based normative sample from 4 to 18 years: the NIH MRI Study of Normal Brain Development. Cerebral cortex. 2012;22(1):1–12. Epub 2011/05/27. 10.1093/cercor/bhr018 21613470 PMC3236790

[41] Wierenga L, Langen M, Ambrosino S, van Dijk S, Oranje B, Durston S. Typical development of basal ganglia, hippocampus, amygdala and cerebellum from age 7 to 24. NeuroImage. 2014;96:67–72. Epub 2014/04/08. 10.1016/j.neuroimage.2014.03.072 . 24705201

[42] Shaw P, Malek M, Watson B, Greenstein D, de Rossi P, Sharp W. Trajectories of cerebral cortical development in childhood and adolescence and adult attention-deficit/hyperactivity disorder. Biological psychiatry. 2013;74(8):599–606. Epub 2013/06/04. 10.1016/j.biopsych.2013.04.007 . 23726514 PMC5922431

[43] Greven CU, Bralten J, Mennes M, O'Dwyer L, van Hulzen KJ, Rommelse N, et al Developmentally stable whole-brain volume reductions and developmentally sensitive caudate and putamen volume alterations in those with attention-deficit/hyperactivity disorder and their unaffected siblings. JAMA psychiatry. 2015;72(5):490–9. Epub 2015/03/19. 10.1001/jamapsychiatry.2014.3162 . 25785435

[44] Shaw P, Eckstrand K, Sharp W, Blumenthal J, Lerch JP, Greenstein D, et al Attention-deficit/hyperactivity disorder is characterized by a delay in cortical maturation. Proceedings of the National Academy of Sciences of the United States of America. 2007;104(49):19649–54. Epub 2007/11/21. 10.1073/pnas.0707741104 18024590 PMC2148343

[45] Chein J, Albert D, O'Brien L, Uckert K, Steinberg L. Peers increase adolescent risk taking by enhancing activity in the brain's reward circuitry. Dev Sci. 2011;14(2):F1–10. Epub 2011/04/19. 10.1111/j.1467-7687.2010.01035.x 21499511 PMC3075496

[46] Hirshfeld DR, Biederman J, Brody L, Faraone SV, Rosenbaum JF. Associations between expressed emotion and child behavioral inhibition and psychopathology: a pilot study. Journal of the American Academy of Child and Adolescent Psychiatry. 1997;36:205–13. 10.1097/00004583-199702000-00011 . 9031573

[47] Asarnow JR, Tompson M, Hamilton EB, Goldstein MJ, Guthrie D. Family-expressed emotion, childhood-onset depression, and childhood-onset schizophrenia spectrum disorders: is expressed emotion a nonspecific correlate of child psychopathology or a specific risk factor for depression? Journal of abnormal child psychology. 1994;22(2):129–46. Epub 1994/04/01. . 8064026 10.1007/BF02167896

[48] Burt SA, Klump KL. Prosocial peer affiliation suppresses genetic influences on non-aggressive antisocial behaviors during childhood. Psychological medicine. 2014;44(4):821–30. Epub 2013/05/11. 10.1017/S0033291713000974 23659437 PMC3749251

[49] Hale WW 3rd, Keijsers L, Klimstra TA, Raaijmakers QA, Hawk S, Branje SJ, et al How does longitudinally measured maternal expressed emotion affect internalizing and externalizing symptoms of adolescents from the general community? Journal of Child Psychology and Psychiatry. 2011;52(11):1174–83. Epub 2011/03/16. 10.1111/j.1469-7610.2011.02400.x . 21401595

[50] Cartwright KL, Bitsakou P, Daley D, Gramzow RH, Psychogiou L, Simonoff E, et al Disentangling child and family influences on maternal expressed emotion toward children with attention-deficit/hyperactivity disorder. Journal of the American Academy of Child and Adolescent Psychiatry. 2011;50:1042–53. 10.1016/j.jaac.2011.07.006 . 21961778

[51] Hou J, Chen Z, Natsuaki MN, Li X, Yang X, Zhang J, et al A longitudinal investigation of the associations among parenting, deviant peer affiliation, and externalizing behaviors: a monozygotic twin differences design. Twin research and human genetics: the official journal of the International Society for Twin Studies. 2013;16(3):698–706. Epub 2013/05/11. 10.1017/thg.2013.24 . 23659853

[52] Gizer IR, Ficks C, Waldman ID. Candidate gene studies of ADHD: a meta-analytic review. Human genetics. 2009;126(1):51–90. Epub 2009/06/10. 10.1007/s00439-009-0694-x . 19506906

[53] Caylak E. Biochemical and genetic analyses of childhood attention deficit/hyperactivity disorder. American journal of medical genetics Part B, Neuropsychiatric genetics: the official publication of the International Society of Psychiatric Genetics. 2012;159B(6):613–27. Epub 2012/07/25. 10.1002/ajmg.b.32077 . 22825876

[54] Belsky J, Pluess M. Beyond diathesis stress: differential susceptibility to environmental influences. Psychological bulletin. 2009;135(6):885–908. Epub 2009/11/04. 10.1037/a0017376 . 19883141

[55] Brookes KJ, Mill J, Guindalini C, Curran S, Xu X, Knight J, et al A common haplotype of the dopamine transporter gene associated with attention-deficit/hyperactivity disorder and interacting with maternal use of alcohol during pregnancy. Arch Gen Psychiatry. 2006;63(1):74–81. Epub 2006/01/04. 10.1001/archpsyc.63.1.74 . 16389200

[56] von Rhein D, Mennes M, van Ewijk H, Groenman AP, Zwiers MP, Oosterlaan J, et al The NeuroIMAGE study: a prospective phenotypic, cognitive, genetic and MRI study in children with attention-deficit/hyperactivity disorder. Design and descriptives. European child & adolescent psychiatry. 2015;24(3):265–81. Epub 2014/07/12. 10.1007/s00787-014-0573-4 . 25012461

[57] Kaufman J, Birmaher B, Brent D, Rao U, Flynn C, Moreci P, et al Schedule for Affective Disorders and Schizophrenia for School-Age Children-Present and Lifetime Version (K-SADS-PL): initial reliability and validity data. Journal of the American Academy of Child and Adolescent Psychiatry. 1997;36(7):980–8. Epub 1997/07/01. 10.1097/00004583-199707000-00021 . 9204677

[58] Wechsler D. WISC-III Handleiding. London: The Psychological Corperation; 2002.

[59] Wechsler D. WAIS-III Nederlandstalige bewerking Technische handleiding. London: The Psychological Corperation; 2000.

[60] Brown GW. The Measurement of Family Activities and Relationships: A Methodological Study. Hum Relations. 1966;19(3):241–63. 10.1177/001872676601900301

[61] Sonuga-Barke EJ, Oades RD, Psychogiou L, Chen W, Franke B, Buitelaar J, et al Dopamine and serotonin transporter genotypes moderate sensitivity to maternal expressed emotion: the case of conduct and emotional problems in attention deficit/hyperactivity disorder. Journal of Child Psychology and Psychiatry. 2009;50(9):1052–63. Epub 2009/06/06. 10.1111/j.1469-7610.2009.02095.x . 19490304

[62] Richards JS, Arias Vásquez A, Rommelse NNJ, Oosterlaan J, Hoekstra PJ, Franke B, et al A Follow-Up Study of Maternal Expressed Emotion Toward Children With Attention-Deficit/Hyperactivity Disorder: Relation with Severity and Persistence of ADHD and Comorbidity. Journal of the American Academy of Child and Adolescent Psychiatry. 2014;53(3):311–9. 10.1016/j.jaac.2013.11.011 24565358 PMC4066112

[63] Schachar R, Taylor E, Wieselberg M, Thorley G, Rutter M. Changes in family function and relationships in children who respond to methylphenidate. Journal of the American Academy of Child and Adolescent Psychiatry. 1987;26:728–32. 10.1097/00004583-198709000-00019 . 3667503

[64] Chen W, Taylor E. PACS interview and genetic research in ADHD In: Oades R, editor. Attention-Deficit/Hyperactivity Disorder HKS: Current ideas and ways forward. 1 ed. New York: Nova Science Publishers Inc; 2006 p. 3–20.

[65] Walden B, McGue M, Lacono WG, Burt SA, Elkins I. Identifying shared environmental contributions to early substance use: the respective roles of peers and parents. Journal of abnormal psychology. 2004;113(3):440–50. Epub 2004/08/18. 10.1037/0021-843X.113.3.440 . 15311989

[66] Burt SA, McGue M, Iacono WG. Nonshared environmental mediation of the association between deviant peer affiliation and adolescent externalizing behaviors over time: results from a cross-lagged monozygotic twin differences design. Developmental psychology. 2009;45(6):1752–60. Epub 2009/11/11. 10.1037/a0016687 19899929 PMC2778800

[67] Hicks BM, South SC, Dirago AC, Iacono WG, McGue M. Environmental adversity and increasing genetic risk for externalizing disorders. Arch Gen Psychiatry. 2009;66(6):640–8. Epub 2009/06/03. 10.1001/archgenpsychiatry.2008.554 19487629 PMC2717707

[68] Conners CK, Sitarenios G, Parker JD, Epstein JN. The revised Conners' Parent Rating Scale (CPRS-R): factor structure, reliability, and criterion validity. Journal of abnormal child psychology. 1998;26(4):257–68. Epub 1998/08/13. . 9700518 10.1023/a:1022602400621

[69] Conners CK, Sitarenios G, Parker JD, Epstein JN. Revision and restandardization of the Conners Teacher Rating Scale (CTRS-R): factor structure, reliability, and criterion validity. Journal of abnormal child psychology. 1998;26:279–91. . 9700520 10.1023/a:1022606501530

[70] Conners CK, Erhardt D, Sparrow EP. Conners' Adult ADHD Rating Scales: CAARS. North Tonowanda, NY: Multi-Health Systems; 1999.

[71] Blumenthal JD, Zijdenbos A, Molloy E, Giedd JN. Motion artifact in magnetic resonance imaging: implications for automated analysis. NeuroImage. 2002;16(1):89–92. Epub 2002/04/24. 10.1006/nimg.2002.1076 . 11969320

[72] Patenaude B, Smith SM, Kennedy DN, Jenkinson M. A Bayesian model of shape and appearance for subcortical brain segmentation. NeuroImage. 2011;56(3):907–22. Epub 2011/03/01. 10.1016/j.neuroimage.2011.02.046 21352927 PMC3417233

[73] Brookes K, Xu X, Chen W, Zhou K, Neale B, Lowe N, et al The analysis of 51 genes in DSM-IV combined type attention deficit hyperactivity disorder: association signals in DRD4, DAT1 and 16 other genes. Molecular psychiatry. 2006;11(10):934–53. Epub 2006/08/09. 10.1038/sj.mp.4001869 . 16894395

[74] Xu X, Duman EA, Anney R, Brookes K, Franke B, Zhou K, et al No association between two polymorphisms of the serotonin transporter gene and combined type attention deficit hyperactivity disorder. American journal of medical genetics Part B, Neuropsychiatric genetics: the official publication of the International Society of Psychiatric Genetics. 2008;147B(7):1306–9. Epub 2008/05/03. 10.1002/ajmg.b.30737 . 18452186

[75] Knafo A, Jaffee SR. Gene-environment correlation in developmental psychopathology. Development and psychopathology. 2013;25(1):1–6. Epub 2013/02/13. 10.1017/S0954579412000855 . 23398748

[76] Li J, Ji L. Adjusting multiple testing in multilocus analyses using the eigenvalues of a correlation matrix. Heredity. 2005;95(3):221–7. Epub 2005/08/04. 10.1038/sj.hdy.6800717 . 16077740

[77] Dawson JF. Moderation in Management Research: What, Why, When, and How. J Bus Psychol. 2014;29(1):1–19. 10.1007/s10869-013-9308-7

[78] Chang Z, Lichtenstein P, Asherson PJ, Larsson H. Developmental twin study of attention problems: high heritabilities throughout development. JAMA psychiatry. 2013;70(3):311–8. 10.1001/jamapsychiatry.2013.287 . 23303526

[79] Pingault JB, Viding E, Galera C, Greven CU, Zheng Y, Plomin R, et al Genetic and Environmental Influences on the Developmental Course of Attention-Deficit/Hyperactivity Disorder Symptoms From Childhood to Adolescence. JAMA psychiatry. 2015 10.1001/jamapsychiatry.2015.0469 . 25945901 PMC6328013

[80] Rommel AS, Rijsdijk F, Greven CU, Asherson P, Kuntsi J. A Longitudinal Twin Study of the Direction of Effects between ADHD Symptoms and IQ. PloS one. 2015;10(4):e0124357 10.1371/journal.pone.0124357 25875897 PMC4398424

[81] van der Meer D, Hoekstra PJ, Zwiers M, Mennes M, Schweren LJ, Franke B, et al Brain Correlates of the Interaction Between 5-HTTLPR and Psychosocial Stress Mediating Attention Deficit Hyperactivity Disorder Severity. American journal of psychiatry. 2015;172(8):768–75. 10.1176/appi.ajp.2015.14081035 . 25998280

[82] Thissen AJ, Bralten J, Rommelse NN, Arias-Vasquez A, Greven CU, Heslenfeld D, et al The role of age in association analyses of ADHD and related neurocognitive functioning: A proof of concept for dopaminergic and serotonergic genes. American journal of medical genetics Part B, Neuropsychiatric genetics: the official publication of the International Society of Psychiatric Genetics. 2015 Epub 2015/01/15. 10.1002/ajmg.b.32290 . 25586935

[83] Thompson BL, Stanwood GD. Pleiotropic effects of neurotransmission during development: modulators of modularity. Journal of autism and developmental disorders. 2009;39(2):260–8. Epub 2008/07/24. 10.1007/s10803-008-0624-0 18648918 PMC2777884

[84] Lesch KP, Bengel D, Heils A, Sabol SZ, Greenberg BD, Petri S, et al Association of anxiety-related traits with a polymorphism in the serotonin transporter gene regulatory region. Science. 1996;274(5292):1527–31. Epub 1996/11/29. . 8929413 10.1126/science.274.5292.1527

[85] Congdon E, Canli T. A neurogenetic approach to impulsivity. Journal of personality. 2008;76(6):1447–84. Epub 2008/11/18. 10.1111/j.1467-6494.2008.00528.x 19012655 PMC2913861

[86] Faraone SV, Spencer TJ, Madras BK, Zhang-James Y, Biederman J. Functional effects of dopamine transporter gene genotypes on in vivo dopamine transporter functioning: a meta-analysis. Molecular psychiatry. 2014;19(8):880–9. Epub 2013/09/26. 10.1038/mp.2013.126 . 24061496 PMC13293237

[87] Elia J, Devoto M. ADHD genetics: 2007 update. Curr Psychiatry Rep. 2007;9(5):434–9. Epub 2007/10/05. . 17915085 10.1007/s11920-007-0057-z

[88] Li SC. Neuromodulation and developmental contextual influences on neural and cognitive plasticity across the lifespan. Neuroscience and biobehavioral reviews. 2013;37(9 Pt B):2201–8. Epub 2013/08/27. 10.1016/j.neubiorev.2013.07.019 . 23973556

[89] Hall FS, Perona MT. Have studies of the developmental regulation of behavioral phenotypes revealed the mechanisms of gene-environment interactions? Physiology & behavior. 2012;107(5):623–40. Epub 2012/05/31. 10.1016/j.physbeh.2012.05.014 22643448 PMC3447116

[90] Miguel-Hidalgo JJ. Brain structural and functional changes in adolescents with psychiatric disorders. International journal of adolescent medicine and health. 2013;25(3):245–56. Epub 2013/07/06. 10.1515/ijamh-2013-0058 23828425 PMC3936342

[91] Cohen RA, Grieve S, Hoth KF, Paul RH, Sweet L, Tate D, et al Early life stress and morphometry of the adult anterior cingulate cortex and caudate nuclei. Biological psychiatry. 2006;59(10):975–82. Epub 2006/04/18. 10.1016/j.biopsych.2005.12.016 . 16616722

[92] Weigard A, Chein J, Albert D, Smith A, Steinberg L. Effects of anonymous peer observation on adolescents' preference for immediate rewards. Dev Sci. 2014;17(1):71–8. Epub 2013/12/18. 10.1111/desc.12099 24341973 PMC3869036

[93] Teicher MH, Samson JA, Sheu YS, Polcari A, McGreenery CE. Hurtful words: association of exposure to peer verbal abuse with elevated psychiatric symptom scores and corpus callosum abnormalities. American journal of psychiatry. 2010;167(12):1464–71. Epub 2010/07/17. 10.1176/appi.ajp.2010.10010030 20634370 PMC3246683

[94] Cremers H, van Tol MJ, Roelofs K, Aleman A, Zitman FG, van Buchem MA, et al Extraversion is linked to volume of the orbitofrontal cortex and amygdala. PloS one. 2011;6(12):e28421 Epub 2011/12/17. 10.1371/journal.pone.0028421 22174802 PMC3235124

[95] DeYoung CG, Hirsh JB, Shane MS, Papademetris X, Rajeevan N, Gray JR. Testing predictions from personality neuroscience. Brain structure and the big five. Psychological science. 2010;21(6):820–8. Epub 2010/05/04. 10.1177/0956797610370159 20435951 PMC3049165

[96] Taki Y, Thyreau B, Kinomura S, Sato K, Goto R, Wu K, et al A longitudinal study of the relationship between personality traits and the annual rate of volume changes in regional gray matter in healthy adults. Human brain mapping. 2013;34(12):3347–53. Epub 2012/07/19. 10.1002/hbm.22145 . 22807062 PMC6869938

[97] Bjornebekk A, Fjell AM, Walhovd KB, Grydeland H, Torgersen S, Westlye LT. Neuronal correlates of the five factor model (FFM) of human personality: Multimodal imaging in a large healthy sample. NeuroImage. 2013;65:194–208. Epub 2012/10/16. 10.1016/j.neuroimage.2012.10.009 . 23063449

[98] <Cartwright et al. - 2011—Disentangling child and family influences on maternal expressed emotion toward children with attention-deficit.pdf>. 10.1016/j.jaac.2011.07.006 21961778

[99] Arnold LE, Hurt E, Lofthouse N. Attention-deficit/hyperactivity disorder: dietary and nutritional treatments. Child and adolescent psychiatric clinics of North America. 2013;22(3):381–402, v. Epub 2013/06/29. 10.1016/j.chc.2013.03.001 . 23806311

[100] Hoza B. Peer functioning in children with ADHD. Journal of pediatric psychology. 2007;32(6):655–63. Epub 2007/06/09. 10.1093/jpepsy/jsm024 . 17556400

[101] Tarantino N, Tully EC, Garcia SE, South S, Iacono WG, McGue M. Genetic and environmental influences on affiliation with deviant peers during adolescence and early adulthood. Developmental psychology. 2014;50(3):663–73. Epub 2013/09/11. 10.1037/a0034345 24015689 PMC4249678

[102] Ring HA, Serra-Mestres J. Neuropsychiatry of the basal ganglia. Journal of neurology, neurosurgery, and psychiatry. 2002;72(1):12–21. 11784818 10.1136/jnnp.72.1.12 PMC1737705

