# Supplementary material for: Developmentally Sensitive Interaction Effects of Genes and the Social Environment on Total and Subcortical Brain Volumes
Source: PLoS One. 2016 May 24;11(5):e0155755. doi: 10.1371/journal.pone.0155755 (PMC4878752; doi:10.1371/journal.pone.0155755)
Supplement: S1 Table — Pearson correlation analyses were performed for all variables, except for correlations with peer affiliation and ADHD severity, for which Spearman correlations analyses were performed. a 9-repeat allele present or absent; b short allele present or absent; c 7-repeat allele present or absent short allele present or absent. * Significant at p ≤ .05 ** Significant at p ≤ .01 (DOCX) [file pone.0155755.s001.docx]

**S1 Table. Correlation analyses between environmental measures, maternal or adolescent’s genotype, brain volumes and ADHD severity.**

|  |  | 1 | 2 | 3 | 4 | 5 | 6 | 7 | 8 | 9 | 10 | 11 | 12 | 13 | 14 | 15 | 16 | 17 | 18 |
| --- | --- | --- | --- | --- | --- | --- | --- | --- | --- | --- | --- | --- | --- | --- | --- | --- | --- | --- | --- |
| 1 | Positive peer affiliation |  |  |  |  |  |  |  |  |  |  |  |  |  |  |  |  |  |  |
| 2 | Deviant peer affiliation | -.14^**^ |  |  |  |  |  |  |  |  |  |  |  |  |  |  |  |  |  |
| 3 | Maternal warmth | .03 | -.07 |  |  |  |  |  |  |  |  |  |  |  |  |  |  |  |  |
| 4 | Maternal criticism | .03 | .12^*^ | -.51^**^ |  |  |  |  |  |  |  |  |  |  |  |  |  |  |  |
| 5 | Adolescent *DAT1^a^* | .04 | -.05 | .11^*^ | -.12^*^ |  |  |  |  |  |  |  |  |  |  |  |  |  |  |
| 6 | Adolescent *5-HTT ^b^* | .01 | -.01 | .11^*^ | -.07 | .05 |  |  |  |  |  |  |  |  |  |  |  |  |  |
| 7 | Adolescent *DRD4^c^* | -.01 | -.07 | .11^*^ | -.06 | .01 | -.02 |  |  |  |  |  |  |  |  |  |  |  |  |
| 8 | Maternal *DAT1^a^* | .02 | -.05 | .15^**^ | -.08 | -.48^**^ | -.05 | .10^**^ |  |  |  |  |  |  |  |  |  |  |  |
| 9 | Maternal *5-HTT ^b^* | -.04 | -.04 | .04 | .00 | -.06 | -.35^**^ | .00 | -.01 |  |  |  |  |  |  |  |  |  |  |
| 10 | Maternal *DRD4 ^c^* | -.05 | -.01 | .10 | -.01 | .06 | .01 | -.43^**^ | .06 | .06 |  |  |  |  |  |  |  |  |  |
| 11 | Total Brain | -.06 | .16^**^ | -.02 | -.03 | .06 | -.05 | .00 | -.02 | .02 | -.03 |  |  |  |  |  |  |  |  |
| 12 | Gray matter | -.08^*^ | .03 | .00 | -.06 | .08^*^ | -.04 | .00 | -.02 | .00 | -.01 | .91^**^ |  |  |  |  |  |  |  |
| 13 | Left caudate | -.05 | -.06 | .02 | .01 | -.04 | -.03 | -.03 | .00 | .07 | .02 | .58^**^ | .53^**^ |  |  |  |  |  |  |
| 14 | Right caudate | -.04 | -.05 | .02 | .03 | -.03 | -.03 | -.05 | .02 | .07 | .05 | .58^**^ | .54^**^ | .93^**^ |  |  |  |  |  |
| 15 | Total caudate | -.04 | -.05 | .02 | .02 | -.04 | -.03 | -.04 | .01 | .07 | .04 | .59^**^ | .54^**^ | .98^**^ | .98^**^ |  |  |  |  |
| 16 | Left putamen | -.08^*^ | .10^**^ | -.04 | .00 | .04 | -.04 | -.02 | -.01 | .07 | -.02 | .66^**^ | .53^**^ | .53^**^ | .53^**^ | .54^**^ |  |  |  |
| 17 | Right putamen | -.08^*^ | .13^**^ | -.05 | -.04 | .03 | -.05 | -.03 | -.01 | .08^*^ | -.02 | .68^**^ | .54^**^ | .53^**^ | .53^**^ | .54^**^ | .94^**^ |  |  |
| 18 | Total putamen | -.08^*^ | .12^**^ | -.05 | -.02 | .04 | -.04 | -.03 | -.01 | .07 | -.02 | .68^**^ | .54^**^ | .54^**^ | .54^**^ | .55^**^ | .98^**^ | .98^**^ |  |
| 19 | ADHD severity | -.16^**^ | .32^**^ | -.04 | .13^**^ | -.01 | -.02 | -.05 | -.02 | .04 | .05 | .78^*^ | .03 | .00 | .00 | .00 | .08^*^ | .07^*^ | .08^*^ |

|  |  |  |  |  |
| --- | --- | --- | --- | --- |
|  |  |  |  |  |
|  |  |  |  |  |
|  |  |  |  |  |
|  |  |  |  |  |
|  |  |  |  |  |
|  |  |  |  |  |
|  |  |  |  |  |
|  |  |  |  |  |

*Note*: Pearson correlation analyses were performed for all variables, except for correlations with peer affiliation and ADHD severity, for which Spearman correlations analyses were performed. ^a^ 9-repeat allele present (1) or absent (0); ^b^ short allele present (1) or absent (0); ^c^ 7-repeat allele (1) or absent (0).

* Significant at *p* ≤ .05

** Significant at *p* ≤ .01
