# Supplementary material for: Developmentally Sensitive Interaction Effects of Genes and the Social Environment on Total and Subcortical Brain Volumes
Source: PLoS One. 2016 May 24;11(5):e0155755. doi: 10.1371/journal.pone.0155755 (PMC4878752; doi:10.1371/journal.pone.0155755)
Supplement: S2 Table — EE = expressed emotion; PA = peer affiliation. a Reference group: 10/10 absent; b reference group: short allele absent; c reference group: 7-repeat absent. Findings in bold are significant after correction for multiple testing (p < .014), findings in bold and italic are nominally significant (i.e. not significant after correction for multiple testing; p ≤ .05). All analyses were corrected for age, gender, and collection site. Additionally, total brain volume was included in analyses of subcortical volumes, and total white matter in analyses of total gray matter as well. (DOCX) [file pone.0155755.s002.docx]

**S2 Table. Mixed model analyses testing the separate main effects of maternal expressed emotion, peer affiliation and plasticity genes on brain volumes.**

|  | Gray matter | | |  | Caudate (T) | | |  | Caudate (L) | | |  | Caudate (R) | | |  | Putamen (T) | | |  | Putamen (L) | | |  | Putamen (R) | | | |
| --- | --- | --- | --- | --- | --- | --- | --- | --- | --- | --- | --- | --- | --- | --- | --- | --- | --- | --- | --- | --- | --- | --- | --- | --- | --- | --- | --- | --- |
|  | ***B*** | ***SE*** | ***p*** |  | ***B*** | ***SE*** | ***p*** |  | ***B*** | ***SE*** | ***p*** |  | ***B*** | ***SE*** | ***p*** |  | ***B*** | ***SE*** | ***p*** |  | ***B*** | ***SE*** | ***p*** |  | ***B*** | ***SE*** | ***p*** |  |
| Main EE effects |  |  |  |  |  |  |  |  |  |  |  |  |  |  |  |  |  |  |  |  |  |  |  |  |  |  |  |  |
| 1 Maternal warmth | .05 | 3.72 | .990 |  | .02 | .05 | .679 |  | .01 | .03 | .683 |  | .01 | .03 | .807 |  | -.04 | .06 | .449 |  | -.02 | .03 | .444 |  | -.03 | .03 | .391 |  |
| 2 Maternal criticism | -2.27 | 3.63 | .532 |  | .06 | .05 | .179 |  | .01 | .02 | .623 |  | .03 | .03 | .166 |  | .01 | .05 | .887 |  | .01 | .03 | .692 |  | -.02 | .03 | .538 |  |
|  |  |  |  |  |  |  |  |  |  |  |  |  |  |  |  |  |  |  |  |  |  |  |  |  |  |  |  |  |
| Main PA effects |  |  |  |  |  |  |  |  |  |  |  |  |  |  |  |  |  |  |  |  |  |  |  |  |  |  |  |  |
| 1 Positive peer affiliation | -.01 | .62 | .984 |  | .00 | .01 | .701 |  | .00 | .00 | .497 |  | .00 | .00 | .964 |  | ***-.02*** | ***.01*** | ***.046*** |  | -.01 | .00 | .114 |  | ***-.01*** | ***.00*** | ***.024*** |  |
| 2 Deviant peer affiliation | -.18 | .58 | .751 |  | ***-.02*** | ***.01*** | ***.017*** |  | **-.01** | **.00** | **.012** |  | ***-.01*** | ***.00*** | ***.031*** |  | .00 | .01 | .606 |  | .00 | .00 | .713 |  | .00 | .00 | .545 |  |
|  |  |  |  |  |  |  |  |  |  |  |  |  |  |  |  |  |  |  |  |  |  |  |  |  |  |  |  |  |
| Main gene effects |  |  |  |  |  |  |  |  |  |  |  |  |  |  |  |  |  |  |  |  |  |  |  |  |  |  |  |  |
| 1 *DAT1*^a^ | 5.63 | 4.80 | .241 |  | ***-.14*** | ***.06*** | ***.021*** |  | ***-.07*** | ***.03*** | ***.015*** |  | ***-.06*** | ***.03*** | ***.043*** |  | .00 | .06 | .960 |  | .00 | .03 | .918 |  | -.01 | .03 | .838 |  |
| 2 *5-HTT*^b^ | -1.18 | 4.78 | .805 |  | .01 | .06 | .865 |  | .01 | .03 | .831 |  | .00 | .03 | .932 |  | -.02 | .06 | .711 |  | .00 | .03 | .897 |  | -.02 | .03 | .528 |  |
| 3 *DRD4*^c^ | -2.45 | 4.89 | .617 |  | -.07 | .06 | .278 |  | -.02 | .03 | .469 |  | -.05 | .03 | .163 |  | -.07 | .07 | .284 |  | -.03 | .04 | .374 |  | -.04 | .03 | .207 |  |

*Note:* EE=expressed emotion; PA=peer affiliation. ^a^ Reference group: 10/10 absent; ^b^ reference group: short allele absent; ^c^ reference group: 7-repeat absent. Findings in bold are significant after correction for multiple testing (*p* < .014), findings in bold and italic are nominally significant (i.e. not significant after correction for multiple testing; *p* ≤ .05). All analyses were corrected for age, gender, and collection site. Additionally, total brain volume was included in analyses of subcortical volumes, and total white matter in analyses of total gray matter as well.
