# Supplementary material for: Developmentally Sensitive Interaction Effects of Genes and the Social Environment on Total and Subcortical Brain Volumes
Source: PLoS One. 2016 May 24;11(5):e0155755. doi: 10.1371/journal.pone.0155755 (PMC4878752; doi:10.1371/journal.pone.0155755)
Supplement: S3 Table — PA = peer affiliation. a Reference group: 10/10 absent; b reference group: short allele absent; c reference group: 7-repeat absent. Findings in bold are significant after correction for multiple testing (p < .014), findings in bold and italic are nominally significant (i.e., not significant after correction for multiple testing; p ≤ .05). All analyses were corrected for age, gender, and collection site. Additionally, total brain volume was included in analyses of subcortical volumes, and total white matter in analyses of total gray matter as well. In each model 2- and 3-way interactions with age or age2 were tested and removed when not significant (p > .05) or nominally significant (p ≥ .014). (DOCX) [file pone.0155755.s003.docx]

**S3 Table. Mixed model analyses testing interaction effects between plasticity genes and maternal expressed emotion or peer affiliation on brain volumes.**

|  |  | Gray matter | | |  | Caudate (T) | | |  | Caudate (L) | | |  | Caudate (R) | | |  | Putamen (T) | | |  | Putamen (L) | | |  | Putamen (R) | | |
| --- | --- | --- | --- | --- | --- | --- | --- | --- | --- | --- | --- | --- | --- | --- | --- | --- | --- | --- | --- | --- | --- | --- | --- | --- | --- | --- | --- | --- |
|  |  | ***B*** | ***SE*** | ***p*** |  | ***B*** | ***SE*** | ***p*** |  | ***B*** | ***SE*** | ***p*** |  | ***B*** | ***SE*** | ***p*** |  | ***B*** | ***SE*** | ***p*** |  | ***B*** | ***SE*** | ***p*** |  | ***B*** | ***SE*** | ***p*** |
| 1 | Maternal warmth | -2.38 | 4.92 | .629 |  | .01 | .06 | .867 |  | .00 | .03 | .888 |  | .01 | .03 | .850 |  | -.01 | .07 | .834 |  | -.01 | .04 | .816 |  | -.01 | .03 | .856 |
|  | *DAT1*^a^ | 5.10 | 6.87 | .458 |  | -.14 | .09 | .111 |  | -.07 | .04 | .132 |  | -.07 | .05 | .115 |  | -.19 | .10 | .054 |  | -.07 | .05 | .149 |  | ***-.11*** | ***.05*** | ***.022*** |
|  | *DAT1**warmth | 3.44 | 8.18 | .675 |  | .06 | .10 | .551 |  | .03 | .05 | .580 |  | .03 | .05 | .544 |  | -.05 | .11 | .628 |  | -.02 | .06 | .750 |  | -.03 | .06 | .545 |
|  |  |  |  |  |  |  |  |  |  |  |  |  |  |  |  |  |  |  |  |  |  |  |  |  |  |  |  |  |
| 2 | Maternal criticism | -2.99 | 4.82 | .535 |  | .09 | .06 | .125 |  | .03 | .03 | .301 |  | .06 | .03 | .056 |  | .01 | .07 | .915 |  | .01 | .04 | .759 |  | .00 | .03 | .890 |
|  | *DAT1*^a^ | 4.77 | 6.88 | .489 |  | -.12 | .09 | .149 |  | -.06 | .04 | .166 |  | -.06 | .05 | .160 |  | ***-.19*** | ***.10*** | ***.048*** |  | -.08 | .05 | .129 |  | ***-.11*** | ***.05*** | ***.021*** |
|  | *DAT1**criticism | 3.39 | 8.28 | .682 |  | -.11 | .10 | .297 |  | -.04 | .05 | .498 |  | -.07 | .05 | .184 |  | -.04 | .11 | .694 |  | -.03 | .06 | .636 |  | -.01 | .06 | .826 |
|  | Age |  |  |  |  |  |  |  |  |  |  |  |  |  |  |  |  | .00 | .02 | .827 |  | .00 | .01 | .640 |  | .00 | .01 | .996 |
|  | Age*Criticism |  |  |  |  |  |  |  |  |  |  |  |  |  |  |  |  | ***-.05*** | ***.02*** | ***.016*** |  | ***-.02*** | ***.01*** | ***.016*** |  | ***-.02*** | ***.01*** | ***.022*** |
|  | Age**DAT1* |  |  |  |  |  |  |  |  |  |  |  |  |  |  |  |  | .00 | .03 | .976 |  | .00 | .02 | .992 |  | .00 | .01 | .939 |
|  | Age**DAT1**criticism |  |  |  |  |  |  |  |  |  |  |  |  |  |  |  |  | **.09** | **.03** | **.005** |  | **.05** | **.02** | **.009** |  | **.05** | **.02** | **.006** |
|  |  |  |  |  |  |  |  |  |  |  |  |  |  |  |  |  |  |  |  |  |  |  |  |  |  |  |  |  |
| 3 | Positive peer affiliation | -0.06 | 0.80 | .945 |  | .00 | .01 | .856 |  | .00 | .00 | .962 |  | .00 | .01 | .692 |  | -.01 | .01 | .151 |  | -.01 | .01 | .201 |  | -.01 | .01 | .139 |
|  | *DAT1*^a^ | 6.52 | 4.89 | .183 |  | **-.15** | **.06** | **.014** |  | **-.08** | **.03** | **.010** |  | ***-.07*** | ***.03*** | ***.030*** |  | -.02 | .07 | .757 |  | -.01 | .03 | .834 |  | -.01 | .03 | .692 |
|  | *DAT1**Positive PA | -0.07 | 1.36 | .958 |  | -.01 | .02 | .527 |  | -.01 | .01 | .506 |  | .00 | .01 | .590 |  | -.01 | .02 | .538 |  | .00 | .01 | .653 |  | -.01 | .01 | .457 |
|  |  |  |  |  |  |  |  |  |  |  |  |  |  |  |  |  |  |  |  |  |  |  |  |  |  |  |  |  |
| 4 | Deviant peer affiliation | 0.18 | 0.70 | .801 |  | ***-.02*** | ***.01*** | ***.023*** |  | ***-.01*** | ***.00*** | ***.015*** |  | ***-.01*** | ***.00*** | ***.044*** |  | .01 | .01 | .204 |  | .01 | .00 | .270 |  | .01 | .00 | .185 |
|  | *DAT1*^a^ | 16.37 | 15.04 | .277 |  | -.13 | .19 | .479 |  | -.07 | .09 | .452 |  | -.06 | .10 | .534 |  | .22 | .20 | .273 |  | .10 | .10 | .330 |  | .11 | .10 | .268 |
|  | *DAT1**Deviant PA | -0.84 | 1.11 | .451 |  | .00 | .01 | .919 |  | .00 | .01 | .913 |  | .00 | .01 | .936 |  | -.02 | .01 | .203 |  | -.01 | .01 | .279 |  | -.01 | .01 | .184 |
|  |  |  |  |  |  |  |  |  |  |  |  |  |  |  |  |  |  |  |  |  |  |  |  |  |  |  |  |  |
| 1 | Maternal warmth | -0.27 | 6.55 | .968 |  | -.03 | .08 | .684 |  | -.01 | .04 | .779 |  | -.02 | .04 | .634 |  | -.09 | .09 | .351 |  | -.03 | .05 | .494 |  | -.05 | .05 | .261 |
|  | *5-HTT*^b^ | -9.69 | 6.77 | .153 |  | .07 | .09 | .383 |  | .05 | .04 | .280 |  | .03 | .05 | .543 |  | .02 | .10 | .863 |  | .02 | .05 | .675 |  | -.01 | .05 | .907 |
|  | *5-HTT**warmth | 0.89 | 8.00 | .911 |  | .08 | .10 | .435 |  | .03 | .05 | .561 |  | .05 | .05 | .370 |  | .07 | .11 | .543 |  | .02 | .06 | .698 |  | .04 | .06 | .424 |
|  |  |  |  |  |  |  |  |  |  |  |  |  |  |  |  |  |  |  |  |  |  |  |  |  |  |  |  |  |
| 2 | Maternal criticism | -2.37 | 5.82 | .685 |  | .11 | .07 | .133 |  | .04 | .04 | .246 |  | .06 | .04 | .089 |  | .04 | .08 | .642 |  | .03 | .04 | .421 |  | .00 | .04 | .921 |
|  | *5-HTT*^b^ | -9.99 | 6.72 | .138 |  | .08 | .08 | .319 |  | .05 | .04 | .238 |  | .03 | .05 | .457 |  | .01 | .10 | .939 |  | .02 | .05 | .708 |  | -.01 | .05 | .795 |
|  | *5-HTT**criticism | -0.38 | 7.45 | .960 |  | -.07 | .09 | .419 |  | -.03 | .05 | .519 |  | -.04 | .05 | .378 |  | -.06 | .10 | .538 |  | -.04 | .05 | .473 |  | -.03 | .05 | .625 |
|  |  |  |  |  |  |  |  |  |  |  |  |  |  |  |  |  |  |  |  |  |  |  |  |  |  |  |  |  |
| 3 | Positive peer affiliation | -0.72 | 1.05 | .493 |  | -.02 | .01 | .135 |  | -.01 | .01 | .160 |  | -.01 | .01 | .141 |  | -.01 | .01 | .501 |  | .00 | .01 | .989 |  | -.01 | .01 | .194 |
|  | *5-HTT*^b^ | -1.79 | 4.84 | .712 |  | .01 | .06 | .883 |  | .00 | .03 | .891 |  | .00 | .03 | .903 |  | -.04 | .06 | .527 |  | -.01 | .03 | .714 |  | -.03 | .03 | .391 |
|  | *5-HTT**Positive PA | 0.96 | 1.29 | .460 |  | .03 | .02 | .107 |  | .01 | .01 | .217 |  | .02 | .01 | .062 |  | -.01 | .02 | .449 |  | -.01 | .01 | .209 |  | .00 | .01 | .875 |
|  |  |  |  |  |  |  |  |  |  |  |  |  |  |  |  |  |  |  |  |  |  |  |  |  |  |  |  |  |
| 4 | Deviant peer affiliation | -0.67 | 0.82 | .413 |  | -.01 | .01 | .179 |  | -.01 | .01 | .087 |  | -.01 | .01 | .345 |  | .00 | .01 | .714 |  | .00 | .01 | .828 |  | .00 | .01 | .637 |
|  | *5-HTT*^b^ | -10.64 | 14.22 | .454 |  | .12 | .17 | .479 |  | .03 | .09 | .765 |  | .10 | .09 | .307 |  | -.05 | .18 | .800 |  | -.03 | .10 | .786 |  | -.02 | .09 | .801 |
|  | *5-HTT**Deviant PA | 0.74 | 1.02 | .471 |  | -.01 | .01 | .458 |  | .00 | .01 | .769 |  | -.01 | .01 | .272 |  | .00 | .01 | .963 |  | .00 | .01 | .870 |  | .00 | .01 | .966 |
|  |  |  |  |  |  |  |  |  |  |  |  |  |  |  |  |  |  |  |  |  |  |  |  |  |  |  |  |  |
| 1 | Maternal warmth | -5.20 | 7.56 | .492 |  | .00 | .06 | .977 |  | .00 | .03 | .958 |  | .00 | .03 | .933 |  | -.04 | .07 | .523 |  | -.03 | .03 | .421 |  | -.01 | .03 | .674 |
|  | *DRD4*^c^ | -0.18 | 11.56 | .988 |  | -.06 | .09 | .484 |  | -.03 | .04 | .539 |  | -.03 | .05 | .453 |  | -.07 | .10 | .498 |  | -.03 | .05 | .539 |  | -.04 | .05 | .466 |
|  | *DRD4**warmth | 14.13 | 14.21 | .321 |  | .10 | .11 | .356 |  | .04 | .05 | .479 |  | .06 | .06 | .295 |  | .02 | .12 | .881 |  | .05 | .06 | .481 |  | -.03 | .06 | .662 |
|  |  |  |  |  |  |  |  |  |  |  |  |  |  |  |  |  |  |  |  |  |  |  |  |  |  |  |  |  |
| 2 | Maternal criticism | -1.31 | 4.36 | .764 |  | .05 | .05 | .368 |  | .01 | .03 | .707 |  | .04 | .03 | .184 |  | .02 | .06 | .768 |  | .03 | .03 | .432 |  | -.01 | .03 | .816 |
|  | *DRD4*^c^ | 0.77 | 6.80 | .910 |  | -.05 | .09 | .585 |  | -.02 | .04 | .628 |  | -.03 | .05 | .561 |  | -.07 | .10 | .455 |  | -.03 | .05 | .529 |  | -.04 | .05 | .397 |
|  | *DRD4**criticism | -3.38 | 7.96 | .671 |  | .03 | .10 | .727 |  | .04 | .05 | .451 |  | .00 | .05 | .964 |  | -.07 | .11 | .544 |  | -.05 | .06 | .358 |  | -.01 | .06 | .816 |
|  |  |  |  |  |  |  |  |  |  |  |  |  |  |  |  |  |  |  |  |  |  |  |  |  |  |  |  |  |
| 3 | Positive peer affiliation | 0.14 | 0.79 | .862 |  | .00 | .01 | .824 |  | .00 | .00 | .787 |  | .00 | .01 | .490 |  | -.01 | .01 | .195 |  | -.01 | .01 | .323 |  | -.01 | .01 | .167 |
|  | *DRD4*^c^ | -2.18 | 4.95 | .660 |  | -.06 | .06 | .350 |  | -.02 | .03 | .610 |  | -.04 | .03 | .193 |  | -.06 | .07 | .389 |  | -.02 | .04 | .518 |  | -.04 | .03 | .234 |
|  | *DRD4**Positive PA | -0.54 | 1.32 | .679 |  | -.01 | .02 | .394 |  | .00 | .01 | .633 |  | -.01 | .01 | .252 |  | -.01 | .02 | .548 |  | .00 | .01 | .583 |  | .00 | .01 | .664 |
|  | Age |  |  |  |  |  |  |  |  |  |  |  |  |  |  |  |  |  |  |  |  |  |  |  |  | -.01 | .01 | .089 |
|  | Age*Positive PA |  |  |  |  |  |  |  |  |  |  |  |  |  |  |  |  |  |  |  |  |  |  |  |  | .00 | .00 | .866 |
|  | Age**DRD4* |  |  |  |  |  |  |  |  |  |  |  |  |  |  |  |  |  |  |  |  |  |  |  |  | .01 | .01 | .301 |
|  | Age**DRD4**Positive PA |  |  |  |  |  |  |  |  |  |  |  |  |  |  |  |  |  |  |  |  |  |  |  |  | **.01** | **.00** | **.012** |
|  |  |  |  |  |  |  |  |  |  |  |  |  |  |  |  |  |  |  |  |  |  |  |  |  |  |  |  |  |
| 4 | Deviant peer affiliation | -0.62 | 0.70 | .378 |  | ***-.02*** | ***.01*** | ***.015*** |  | **-.01** | **.00** | **.009** |  | ***-.01*** | ***.00*** | ***.032*** |  | .01 | .01 | .348 |  | .00 | .00 | .386 |  | .00 | .00 | .335 |
|  | *DRD4*^c^ | -14.29 | 14.59 | .328 |  | -.15 | .18 | .417 |  | -.08 | .09 | .366 |  | -.06 | .10 | .500 |  | .10 | .19 | .602 |  | .06 | .10 | .547 |  | .04 | .10 | .684 |
|  | *DRD4**Deviant PA | .89 | 1.07 | .403 |  | .01 | .01 | .699 |  | .00 | .01 | .535 |  | .00 | .01 | .889 |  | -.01 | .01 | .332 |  | -.01 | .01 | .326 |  | -.01 | .01 | .360 |

*Note:* PA=peer affiliation. ^a^ Reference group: 10/10 absent; ^b^ reference group: short allele absent; ^c^ reference group: 7-repeat absent. Findings in bold are significant after correction for multiple testing (*p* < .014), findings in bold and italic are nominally significant (i.e., not significant after correction for multiple testing; *p* ≤ .05). All analyses were corrected for age, gender, and collection site. Additionally, total brain volume was included in analyses of subcortical volumes, and total white matter in analyses of total gray matter as well. In each model 2- and 3-way interactions with age or age^2^ were tested and removed when not significant (*p* > .05) or nominally significant (*p* ≥ .017).
